# A model of the indirect losses from negative shocks in production and finance

**DOI:** 10.1371/journal.pone.0239293

**Published:** 2020-09-23

**Authors:** Hazem Krichene, Hiroyasu Inoue, Takashi Isogai, Abhijit Chakraborty

**Affiliations:** 1 Potsdam Institute for Climate Impact Research, Potsdam, Germany; 2 Graduate School of Simulation Studies, University of Hyogo, Kobe, Japan; 3 Graduate School of Social Sciences, Tokyo Metropolitan University, Tokyo, Japan; 4 Complexity Science Hub Vienna, Vienna, Austria; 5 Advanced Systems Analysis, International Institute for Applied Systems Analysis (IIASA), Laxenburg, Austria; Beijing University of Technology, CHINA

## Abstract

Economies are frequently affected by natural disasters and both domestic and overseas financial crises. These events disrupt production and cause multiple other types of economic losses, including negative impacts on the banking system. Understanding the transmission mechanism that causes various negative second-order post-catastrophe effects is crucial if policymakers are to develop more efficient recovery strategies. In this work, we introduce a credit-based adaptive regional input-output (ARIO) model to analyse the effects of disasters and crises on the supply chain and bank-firm credit networks. Using real Japanese networks and the exogenous shocks of the 2008 Lehman Brothers bankruptcy and the Great East Japan Earthquake (March 11, 2011), this paper aims to depict how these negative shocks propagate through the supply chain and affect the banking system. The credit-based ARIO model is calibrated using Latin hypercube sampling and the design of experiments procedure to reproduce the short-term (one-year) dynamics of the Japanese industrial production index after the 2008 Lehman Brothers bankruptcy and the 2011 Great East Japan earthquake. Then, through simulation experiments, we identify the chemical and petroleum manufacturing and transport sectors as the most vulnerable Japanese industrial sectors. Finally, the case of the 2011 Great East Japan Earthquake is simulated for Japanese prefectures to understand differences among regions in terms of globally engendered indirect economic losses. Tokyo and Osaka prefectures are the most vulnerable locations because they hold greater concentrations of the above-mentioned vulnerable industrial sectors.

## Introduction

Economies are vulnerable to successive negative shocks, which could be a natural disaster, such as Hurricane Katrina in the U.S. [1] or the 2011 Great East Japan Earthquake in Japan (henceforth, the 2011 Great Earthquake) [2, 3], or an economic or financial crisis, such as the 2007-2008 financial crisis [4, 5] or the Japanese banking crisis [6].

Natural disasters cause economic losses. Usually, it is straightforward to define the direct losses from a disaster, as they are the immediate consequences of the disaster as defined in [7]. However, the shock of the initial damage leads to larger and longer term losses, as discussed in [7], which are defined as indirect losses. The debate in economics over how to efficiently estimate the indirect losses from a disaster remains unresolved. Indeed, due to the complexity of the economic environment, it is difficult to quantify all indirect losses. Indirect losses may be defined to include the modification of consumer behaviour after the disaster (such as their saving-consumption strategy), supply chain disruptions, stock market losses, increased bank credit defaults, rising insurance costs, and a higher level of government expenditures.

Input-output (IO) models are one of the simplest measures of the indirect losses from a natural disaster [8]. The advantage of this approach is that it is modelled on inter-industry links and the production network’s supply and demand structure. Thus, it could easily capture the indirect losses due to the supply shortages. One application is the multi-regional input-output model (MRIO) [9] to evaluate the global effects of a supply chain perturbation induced by a disaster due to economic interdependence between different regions. A recent development is the adaptive regional input-output (ARIO) model introduced in [10, 11]. In fact, compared to the MRIO model, the ARIO model provides two main advantages: i) the ARIO model introduces a temporal dynamics of the economic in the aftermath of a disaster; ii) the ARIO model provides a highest resolution analysis at the firm level. However, the IO approach suffers from rigidity, as it does not allow for the possibility of recovery or an adaptive strategy by agents, which leads to overestimated indirect losses, as explained in [12]. To overcome this well-known limitation of IO models, [13, 14] introduced an autonomous recovery mechanism for firms in the production network after a disaster. These authors create a simulation with their recovery-based ARIO model using the real production network of Japan (2011 data) and successfully reproduce the dynamics of the value added (VA) of the Japanese economy in the aftermath of the 2011 Great Earthquake.

The various versions of the ARIO framework assume that output losses result from interrupted or disrupted production. As the World Economic Forum documents (https://www.weforum.org/agenda/2014/08/natural-disasters-firm-activity-damage-banks), following the 2011 Great Earthquake, 36.5% of firms indicated that they were negatively affected by damage experienced by their suppliers, and 44% stated that they were negatively affected by damage experienced by their customers. Moreover, 11.4% of firms indicated that they were affected by damage experienced by their major lender bank. [15] study the effect of the 1995 Great Hanshin-Awaji earthquake in Japan and show that it weakened the financial capacity of borrowing firms, which deteriorated banks’ loan portfolios (increasing non-performing loans, NPL) and hence reduced their risk-taking capacity. The decline in banks’ lending capacity reduces credit supply, which has a negative impact on borrowing firms’ activities, which is another indirect effect of disasters that reduces the VA of the economy. [16] demonstrate that damage to firms weakens the stability of U.S. banks and reduces their risk performance and credit supply. The authors show that after disasters, banks suffer from higher NPL, a lower return on assets (ROA), and lower equity ratios.

Financial crises are regarded as banking panics caused by liquidity or credit default problems, as described in [4]. In these cases, a domino effect may be observed via the credit defaults of insolvent banks and the contraction of bank lending, which restricts firms’ financial resources. Thus, firms reduce their production and may suffer unexpected losses (feedback effect to the banking system); see [17]. Most research on financial crises examines the domino effect of consecutive bankruptcies to measure the resilience of the economy. [18] consider an artificial bipartite network of banks and bank assets and propose a cascading model that reproduces the bankruptcies of banks in the U.S. during the 2008 financial crisis. [19] improves on this cascading failure approach by considering a bank-firm bipartite network. The author introduces nonlinear interactions between the financial and non-financial sectors. These works consider firms or banks to be either healthy or bankrupt, which means that they fail to measure the indirect losses suffered by the economy. In fact, losses during financial crises are not only related to bankruptcies but can also be reflected in, for example, a reduction in firms’ production, an increased NPL rate, or reduced liquidity. In addition, some countries suffered from the 2008 financial crisis without observing such failure cascades, although those economies did suffer from the systemic crisis. [20] asked why Japan was severely affected by the 2008 financial crisis despite its resilient banking system. The author’s answer is the high dependence of Japanese industries on global trading networks and because Japan is the primary exporter country to emerging Asian economies, which are the primary exporters of final goods to the U.S. and Europe. Accordingly, the financial resources of Japanese firms were negatively affected, which decreased their production capacity, affecting the production network. The transmission channel from the external demand shock to the Japanese stock market and the production network is discussed in [21]. Following the words of the author: ‘*The damaging impact of Japanese export decline to these advanced countries was also exacerbated by Japan’s new trade structure that utilized a substantially regionalized production network*’. Consequently, the negative shock due to the 2008 financial crisis was neither a credit shock nor a domestic banking bankruptcy shock in Japan. Therefore, the 2008 Lehman Brothers bankruptcy could be considered an exogenous shock to the real supply chain in the Japanese economy.

To the best of our knowledge, all existing models that simulate the spread of contagion treat financial crises and natural disasters separately. In a contribution following the conference titled *Crises and Disasters: Measurement and Mitigation of their Human Costs*, [22] discusses several presented papers and highlights the need to examine the strategies adopted by agents during crises and natural disasters and of exploring how public agencies can be more efficient in protecting the wellbeing of households; see also [23] for further discussion of risk management during financial crises and natural disasters. Therefore, the main contribution of this work is that it builds a framework able to measure the indirect economic losses due to financial crises and natural disasters.

This paper proposes a framework based on the ARIO model discussed in [11, 13, 14]. First, we add the modelling of the Japanese credit market based on real bank-firm credit network data, which allows the credit-based ARIO to measure the effect of an exogenous shock to real production on the banking system. Second, we employ Latin hypercube sampling and the design of experiments approach discussed in [24] to calibrate the parameters of the credit-based ARIO model to the cases of the 2008 Lehman Brothers bankruptcy and the 2011 Great Earthquake in Japan. Third, we specify the most vulnerable industrial sectors and the most vulnerable prefectures in Japan.

In the credit-based ARIO, production is represented by the supply chain network of listed Japanese firms, where the weight of links represents the money flows of traded intermediate goods between suppliers and customers. In the credit-based ARIO, a customer should pay its suppliers when purchasing an intermediate good. Credit is represented by the bank-firm network, where each firm has initial loans and deposits with its lending banks. Each firm has an initial balance sheet that evolves following the dynamics of the credit-based ARIO model. A firm purchases intermediate goods with its deposits or, if necessary, short-term bank loans, produces goods following a linear production function, sells intermediate goods to its customers and final goods to households, and increases its bank deposits based on its profits. Following a negative shock, a firm uses a recovery loan supplied by banks connected to the bank-firm network for reconstruction or financial recovery, depending on the nature of the damage. Then, each firm continues its economic process as described previously. A negative shock is modelled by an initial disruption of production. Then, the model measures the effect of the initial shock on the VA of the economy and on the banks’ ratios (NPL and liquidity).

Next section presents the production network of Japanese listed firms and the bank-firm credit network. Then, we introduce the credit-based ARIO model and discuss its assumptions. Thus, another section reports the model initialization, the simulation experiments, and the model calibration using the design of experiments procedure and Latin hypercube sampling. Therefore, we present the experimental simulation results. Finally, last section offers a discussion, concluding remarks and directions for future research.

## Real data and the modelled networks

We present real economic data captured after disaster events. Some of these data will be used to calibrate our model. Then, we present the network data used in the credit-based ARIO model.

### The effects of the 2008 financial crisis and the 2011 Great Earthquake

**Fig 1:**
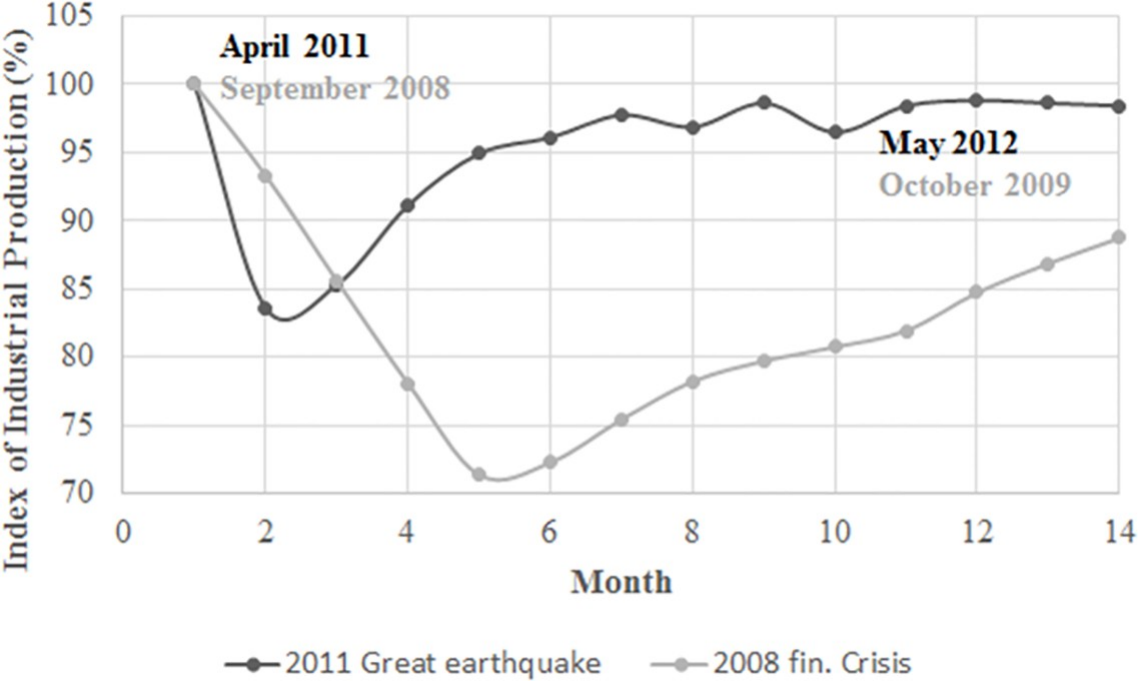
The dynamics of the IIP of Japan after two negative shocks. Lehman Brothers bankruptcy in September 2008 and the 2011 Great Earthquake. The initial IIP is treated as the reference. Source: The Ministry of Economy, Trade and Industry.

**Fig 2:**
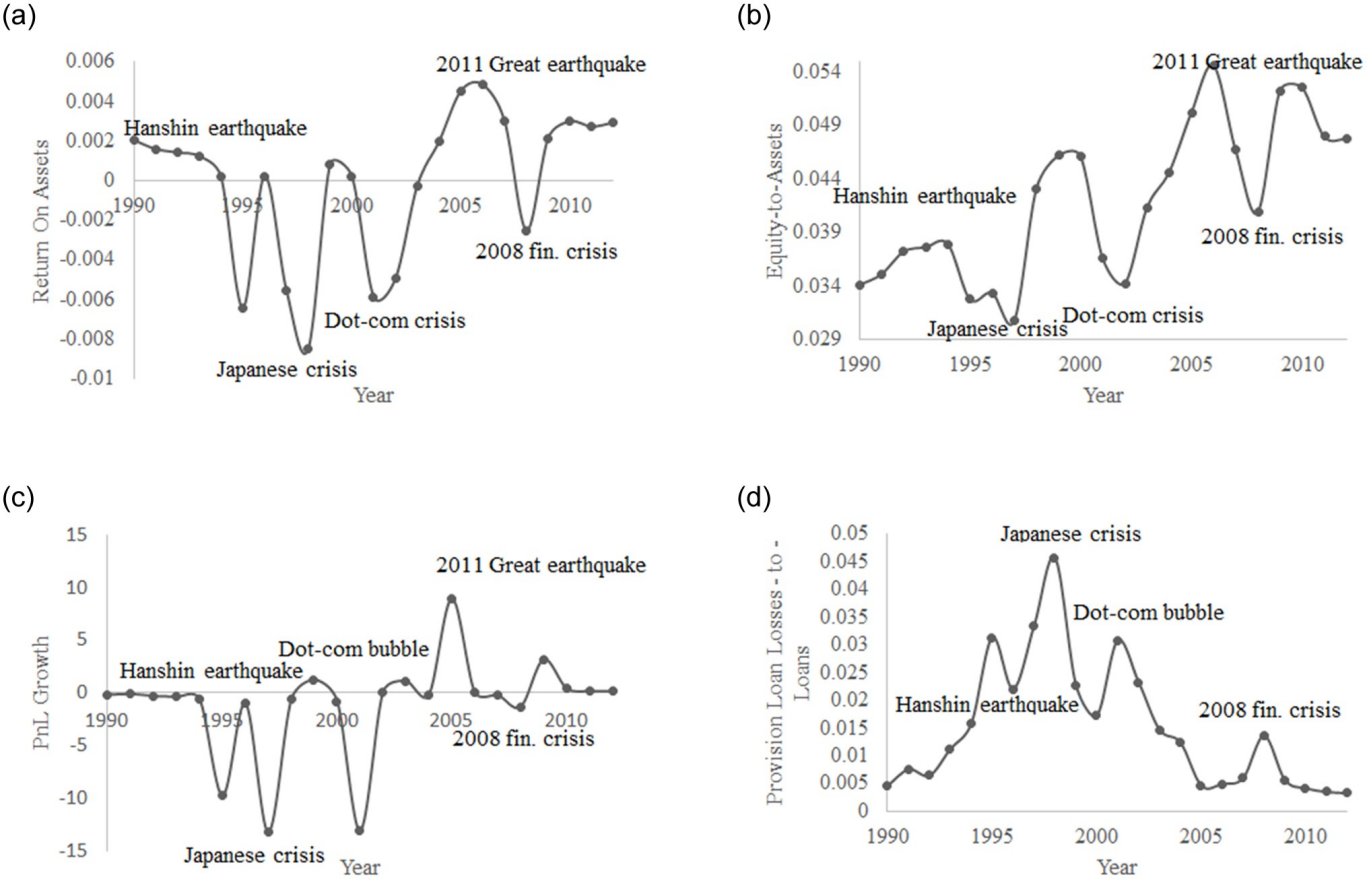
Some risk and performance ratios for Japanese banks from 1990 to 2012. Return on assets (ROA) in Fig 2(a), equity to assets ratio in Fig 2(b), profit and loss (PnL) growth in Fig 2(c) and provision for loan losses (PLL) to loans ratio in Fig 2(d). All negative shocks are characterized by a higher risk level for banks with lower performance. Source: Bank of Japan statistics.

**Fig 3:**
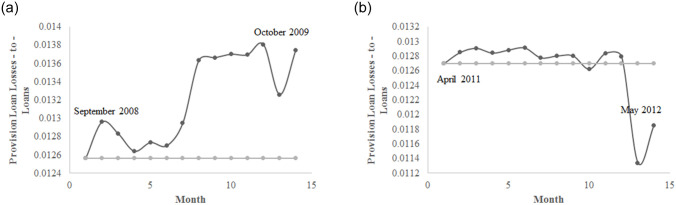
The dynamics of the provision for loan losses to loans ratio. The 2008 Lehman Brothers bankruptcy (a) and the 2011 Great Earthquake (b). The grey line represents the level of NPL before the negative shock. Source: Bank of Japan statistics.

Fig 1 shows the losses in terms of the index of industrial production (IIP) in Japan after the 2008 Lehman Brothers bankruptcy and the 2011 Great Earthquake. The two negative shocks are reflected by an initial crash in production and a progressive recovery. We will attempt to reproduce these IIP dynamics using our model. Fig 2 displays 4 ratios of Japanese banks from 1990 to 2012: ROA in Fig 2(a), the equity to assets ratio in Fig 2(b), profit and loss (PnL) growth in Fig 2(c) and the provision for loan losses (PLL) to loans ratio in Fig 2(d). The evolution of these ratios indicates that during negative shocks (the Hanshin-Awaji earthquake, the Japanese banking crisis, the dot-com crisis, the 2008 financial crisis and the 2011 Great Earthquake), Japanese banks became less stable, exhibiting a lower solvency ratio (Fig 2(b)) and higher loan losses (Fig 2(d)). Moreover, their performance was much lower, as reflected by the decreases in ROA and PnL growth, Fig 2(a) and 2(c). Fig 3(a) depicts the 1-year dynamics of the PLL-to-loans ratio after the 2008 Lehman Brothers bankruptcy, and Fig 3(b) displays the same statistics in the aftermath of the 2011 Great Earthquake. Both figures show a positive effect on the PLL-to-loans ratio, which reflects higher expected NPL. This ratio increased by 9.4% on average following the 2008 economic disaster and only 1.2% following the 2011 natural disaster, which also exhibited a faster recovery.

### The data used in the credit-based ARIO model

**Fig 4:**
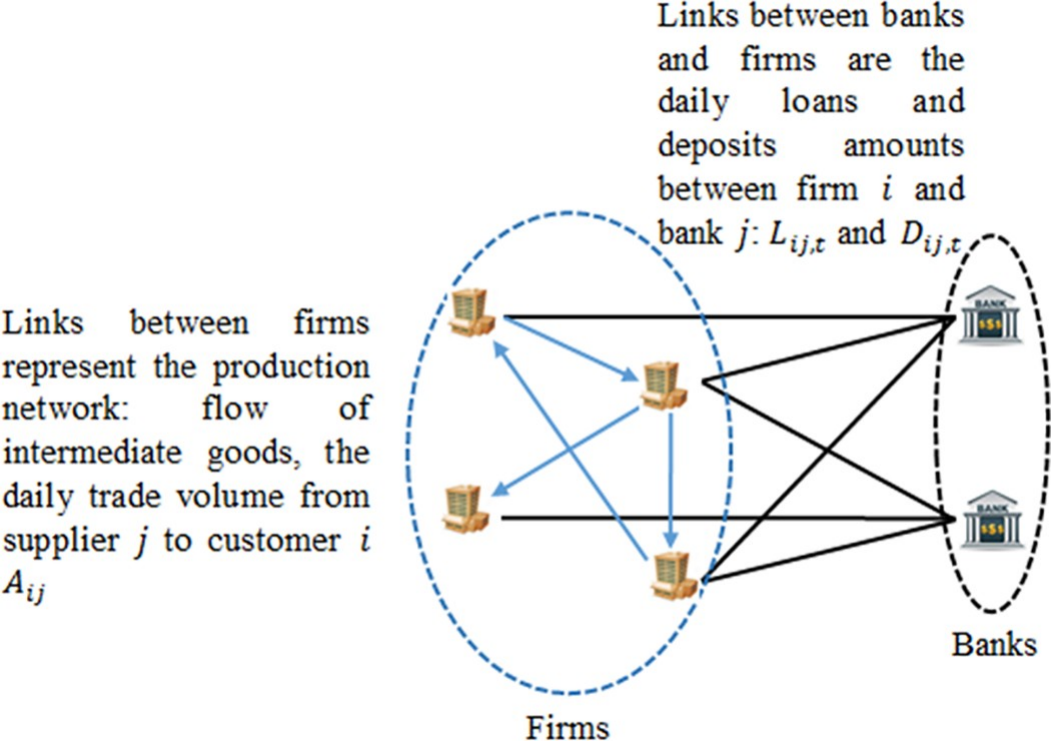
A schematic representation of the production network and the bank-firm network considered in the credit-based ARIO. Links between firms in the production network are weighted by the amount of the daily trading flows of intermediate goods *A*_*ij*_ between firms *i* and *j*. Links between firms and banks are weighted by the daily amounts of loans *L*_*ij*,*t*_ and deposits *D*_*ij*,*t*_ between firm *i* and bank *j*.

Two networks are considered in the credit-based ARIO model: the production network of Japanese listed firms and their corresponding bank-firm network. Fig 4 is a schematic representation of the considered economy. In the following, we present the data and discuss the initialization of the intermediate goods flows and firms’ balance sheets.

The Japanese production network consists of 1,247,521 firms and 5,488,484 distinct links representing customer-supplier trading for the year 2016. Data are collected by Tokyo Shoko Research (TSR), Inc., one of the leading credit research agencies in Japan, and are commercially available. TSR collects information about the 24 major suppliers and clients of each firm through questionnaires. However, the number of suppliers and customers of each firm is not limited to 24 because large firms are designated by many other firms as suppliers or customers. Each firm belongs to an industrial sector. We use the 190 basic sector classifications of the IO tables for Japan in 2016 obtained from the Ministry of Economy, Trade, and Industry. Analysis of the Japanese production network is discussed in several works, such as [25–30].

The other data set used here is the bank-firm network. It represents the lending-borrowing relationships between listed Japanese firms and banks and their annual financial statements. The data set is collected yearly by Nikkei Media Marketing, Inc. and is also commercially available. We use the 2016 bank-firm network and consider the subset of listed firms from the TSR production network. Accordingly, the production network considered in the remainder of this paper has 2,169 firms and 8,841 production trading links, while the considered bank-firm network has 165 banks (including 88 listed banks), 2,169 firms and 18,535 lending-deposit links. Although the number of listed firms is small compared to the total size of the production network, they represent 22% of the total net sales of Japanese firms. A recent analysis of this network is provided in [31].

The production network is initially unweighted, and the bank-firm network has information about loans only (no information on deposits between individual firms and banks is available). Thus, based on the 2016 IO table for Japan and firms’ balance sheets, we calibrate the initial weights of the model as described in the model validation section. A schematic representation of the data structure is given in Fig 16 of Appendix A.

## The credit-based ARIO model

We first describe the model and discuss our assumptions. We then present the details of the framework. The credit-based ARIO implementation is publicly available on Github (The framework can be tested with pretend data. Real data cannot be shared publicly. The credit-based ARIO model is developed under C++ programming language. Github: https://github.com/hazem2410/SNSE). For clarity, Fig 15 (Appendix A) shows a schematic representation of the credit-based ARIO model associated with the different equations discussed hereafter.

### Assumptions and model description

We assume a closed credit-based economy, where agents are firms, banks, and final consumers. Several macroeconomic agent-based models (ABMs) assume, e.g., [33], that the absence of a foreign market makes the model simpler and better able to focus on the spread of negative shocks within the economy. To relax this assumption, we need to model the interactions with foreign economies via international trade. This would capture the effects of local negative shocks on net export activity.

The modelled economy has short-term dynamics (1 year). Thus, we assume that firms keep their commercial partners identified in the real data from before the negative shock and that banks keep their borrowing-lending links with their pre-shock clients. Therefore, firms and banks do not create new connections within 1 year of a shock based on the fact that it is difficult to obtain new trade contracts during economic contractions with a lower level of solvency; see [11]. Moreover, it is assumed in the short run that final consumers maintain the same level of demand for final goods, which is supported by the empirical findings in [32]. This strong assumption should be relaxed to extend the model for the mid- and longer-term impact.

Each firm produces a specific product defined by its industrial sector, i.e., firms from same industrial sectors produce the same intermediate goods. Firm *i* initially produces *P*_*ini*,*i*_ using intermediate goods *A*_*ij*_ from all its suppliers *j*. Customer *i* in the production network purchases a quantity of intermediate goods *A*_*ij*_ on day *t* from its suppliers *j*. Then, customer *i* pays each of its suppliers *j* the amount *A*_*ij*_. Customer *i* pays its obligations to its suppliers out of its total deposits; see Eq 26. If deposits cannot cover production expenses, customer *i* seeks a short-term loan from its banks.

For simplicity and without loss of generality, we suppose that banks are only risk managers, as in [33]. Indeed, our model focuses only on the effects of loan supply and the possible impact of negative shocks on credit default. Thus, banks are homogeneous in their behaviour and are not asset-liability optimizers, i.e., they do not attempt to optimize their credit portfolios. In addition, because we focus only on the supply of loans, the credit market between firms and banks is the only modelled financial market in the economy. Thus, no interbank market is considered, and no central bank is represented in the model. Because of the latter assumption, our model cannot capture the contagion effects between banks, as in the case of a financial crisis (see [34]), which could lead it to underestimate or overestimate the indirect losses related to exogenous negative shocks. However, because we are studying the short-run dynamics post-economic shock on the production network, we could assume that the contagion effect between banks will be delayed in time. Consequently, the absence of an interbank market will not significantly change the conclusions in this framework. To study longer-term economic dynamics, this assumption should be relaxed in future development of the model.

### Firm production after a negative shock

The production process is given as follows: demand for intermediate goods, production process, trading, and inventory dynamics.

#### Demand for intermediate goods

At the beginning of day *t*, regardless of whether the economy has been damaged by a shock, each firm *i* desires a quantity of each intermediate good from its different suppliers *j*. As introduced in [11], the desired quantity is given by:

$$\begin{array}{c} Q_{ij,t}^{d}=A_{ij}\times \frac{P_{i,t-1}^{R}}{P_{ini,i}}+\frac{1}{\tau }\times (n_{i}\times A_{ij}\times \frac{P_{i,t-1}^{R}}{P_{ini,i}}-S_{ij,t}) \end{array}$$ (1)

Eq 1 has the following interpretation: *S*_*ij*,*t*_ is the inventory held by firm *i* of the intermediate good produced by firm *j* on day *t*. Firm *i* seeks to maintain a level of inventory that allows the utilization of good *j* for *n*_*i*_ days. Firms use a very simple forecasting rule, i.e., production at time *t* is predicted to be equal to the realized production at time *t* − 1 denoted by $P_{i,t-1}^{R}$. Accordingly, firm *i* demands consumption of product *j* as the fraction $\frac{P_{i,t-1}^{R}}{P_{ini,i}}$ (fraction between realized and initial production) from its initial input level *A*_*ij*_. Demand is adjusted by the current level of inventory *S*_*ij*,*t*_ and the objective level of inventory, which depends on the number of days *n*_*i*_. The gap between the current and the target inventory is filled gradually by the ratio 1/*τ*. $Q_{i,t}^{D}=\sum_{j}Q_{ij,t}^{d}$ is the aggregate of goods desired by firm *i* on day *t*.

#### Production process

We assume that the full production capacity of firm *i* over one year cannot exceed its initial production *P*_*ini*,*i*_. Accordingly, as in [11], the production capacity of firm *i* on day *t* is defined as:

$$\begin{array}{c} P_{i,t}^{c}=\left(1-\delta_{i,t}\right)\times P_{ini,i} \end{array}$$ (2)

*δ*_*i*,0_ represents the direct damage that affects firm *i*. Direct damage can be physical damage or financial damage. After a natural disaster, damaged firms suffer from the destruction of buildings, machines, and so forth, which is considered physical damage. During a financial crisis, firms face constrained financial resources, which reduces their production capacity. Although Japanese banks were resilient after the 2008 Lehman Brothers bankruptcy, Japanese firms’ production suffered, as explained in [20]. We assume that *δ*_*i*,0_ is the same for all directly affected firms. However, the dynamics of *δ*_*i*,*t*_ depend on the recovery capacity of the company, as will be explained below.

The inventory of intermediate good *s* held by firm *i* on day *t* is given by:

$$\begin{array}{c} S_{i,t}^{s}=\sum_{j\in N_{i}^{s}}S_{ij,t} \end{array}$$ (3)

$N_{i}^{s}$ is the set of suppliers of firm *i* that produce product *s*. In addition, the initial input of the intermediate good *s* is given by:

$$\begin{array}{c} A_{i}^{s}=\sum_{j\in N_{i}^{s}}A_{ij} \end{array}$$ (4)

Therefore, the production of firm *i* on day *t* could be limited by the available quantity of product *s*, which models the indirect effect after the negative shock due to the shortage of supply given by:

$$\begin{array}{c} P_{i,t}^{s}=\frac{S_{i,t}^{s}}{A_{i}^{s}}\times P_{ini,i} \end{array}$$ (5)

Thus, the maximum production based on inventories of products *s*_*i*_ used by firm *i* is given by:

$$\begin{array}{c} P_{i,t}^{\text{max}}=\text{min}\left(P_{i,t}^{c},\underset{s_{i}}{\text{min}}\left(P_{i,t}^{s_{i}}\right)\right) \end{array}$$ (6)

Furthermore, a firm does not produce more than the demand it receives from its customers and final consumers. Final consumers demand a constant aggregate quantity *C*_*i*_. Then, the total received demand is given by:

$$\begin{array}{c} Q_{i,t}^{r}=C_{i}+\sum_{j}Q_{ji,t}^{d}. \end{array}$$ (7)

Finally, the production of firm *i* on day *t* is defined by:

$$\begin{array}{c} P_{i,t}=\text{min}\left(P_{i,t}^{\text{max}},Q_{i,t}^{r}\right) \end{array}$$ (8)

#### Trading process

At each day *t*, firms engage in trade. Suppliers sell intermediate goods, and customers pay their obligations.

#### Selling and rationing

When received demand $Q_{i,t}^{r}$ (see Eq 7) is equal to production *P*_*i*,*t*_, supplier *i* can satisfy all of its client demand. Otherwise, if $Q_{i,t}^{r}>P_{i,t}$, supplier *i* needs to ration goods. We follow the algorithm introduced by [13, 14].

First, supplier *i* calculates the ratio of pre-to-post negative shock orders for each client *j* $\rho_{j,t}=Q_{ji,t}^{d}/A_{ji}$; note that this ratio remains 1 for final consumers. The minimum of the calculated ratios $\rho_{j,t}^{\text{min}}$ is applied to all received demand $Q_{i,t}^{r}$. If the new aggregate demand remains greater than the remaining production capacity, supplier *i* shares its product equally among all its clients. Otherwise, supplier *i* begins by satisfying the demand of all its clients following $\rho_{j,t}^{\text{min}}$. Then, the ratio of each client *j* is updated, $\rho_{j,t}^{*}=\rho_{j,t}-\rho_{j,t}^{\text{min}}$, and the same procedure is applied until the remaining production capacity is equal to 0. At the end of the rationing procedure, firm *i* defines the effective realized demand $Q_{ji,t}^{*}$ and $C_{i}^{*}$. Then, the possible total sales are given as follows:

$$\begin{array}{c} Y_{i,t}^{e}=P_{i,t}=C_{i}^{*}+\sum_{j}Q_{ji,t}^{*} \end{array}$$ (9)

#### Payment process and inventory dynamics

Following the improvement of the ARIO model described in [11, 13, 14], we propose including additional indirect economic losses due to the demand shortage effect. If the total deposits of customer *i*, *D*_*i*,*t*_, are greater than its total realized goods, $Q_{i,t}^{*}=\sum_{j}Q_{ij,t}^{*}$, trading is completed. Otherwise, customer *i* seeks a short-term loan from its banks to cover its funding needs, as will be explained in greater detail below.

If the customer secures bank lending, it can pay its suppliers, and therefore it purchases all demanded goods. Otherwise, the customer should re-define its demand based on its current deposits. Because products are inelastic substitutes, the customer reduces the quantity of all its demanded goods by an equal amount. Let us assume that $\mu_{i,t}^{Q}=\left(Q_{i,t}^{*}-D_{i,t}\right)/Q_{i,t}^{*}$. The final obtained goods *j* are given as $Q_{ij,t}^{F}=\mu_{i,t}^{Q}\times Q_{ij,t}^{*}$. We assume that final consumers are always able to meet their obligations and do not need loans. This could be justified by the empirical findings of [32], who show that households keep their level of consumption during a crisis and are not affected by a shortage in the loan supply because they rely on drawing down liquid assets. This strong assumption is motivated by the short-run dynamics of the model. However, it should be relaxed to extend the model for longer-term dynamics. Consequently, the effective realized sales by supplier j are given by:

$$\begin{array}{c} Y_{j,t}=Y_{j,t}^{e}-\sum_{i}(Q_{ij,t}^{*}-Q_{ij,t}^{F}) \end{array}$$ (10)

Finally, the inventory of product *j* in firm *i* evolves as follows:

$$\begin{array}{c} S_{ij,t+1}=S_{ij,t}+Q_{ij,t}^{F}-A_{ij}\times \frac{P_{i,t}}{P_{ini,i}} \end{array}$$ (11)

The VA generated by each firm is equal to the difference between the used goods and the realized sales. Thus, the VA of the economy is given by:

$$\begin{array}{c} {\text{VA}}_{t}=\sum_{i}[Y_{i,t}-\sum_{j}A_{ij}\times \frac{P_{i,t}}{P_{ini,i}}] \end{array}$$ (12)

### Short-term bank lending

Each firm *i* may seek a loan from its banks depending on its demand $Q_{i,t}^{*}$ and its current deposit level *D*_*i*,*t*_. If deposits cannot cover its expenses, firm *i* seeks a total amount of short-term loans given by:

$$\begin{array}{c} L_{i,t}^{d}=Q_{i,t}^{*}-D_{i,t} \end{array}$$ (13)

The demanded loan $L_{i,t}^{d}$ is divided equally among all its banks from the bank-firm network, i.e., for simplicity, we assume that bank-firm relationships are homogeneous. All short-term loans have the same maturity *T*_*s*_. We further assume that all banks apply the same interest rate given by:

$$\begin{array}{c} r_{i,t}^{s}=\left(1-\frac{Y_{i,t}}{P_{ini,i}}\right)\times r \end{array}$$ (14)

*r* is the market interest rate and is assumed to be constant, *r* = 1%. Following Eq 11, in the pre-shock situation, *Y*_*i*,*t*_ = *P*_*ini*,*i*_, loans are supplied at a 0 interest rate. In an analysis of the short-term lending market, [35] confirms that the interest rate elasticity of loan demand is significant. Thus, we expect that the interest rate increases when demand for loans increases. In the credit-based ARIO model, when firms face greater damage and lower sales *Y*_*i*,*t*_, their deposits decrease because of their lower profits. Thus, these firms increase their loan demand. Accordingly, based on Eq 13, when sales decrease, loan demand and the interest rate on short-term loans both increase. In addition, Eq 14 reflects that the banks in our model follow risk-based pricing for the interest rate, as discussed in [36]. In fact, the more strongly affected firms have lower production, generate less profit and have higher risk of default, which increases the interest rate pricing based on Eq 14.

Banks have access to the financial statements of their clients, which is reflected by the leverage ratio calculated as in [33]:

$$\begin{array}{c} \lambda_{i,t}=\frac{L_{i,t}^{d}+L_{i,t-1}}{E_{i,t-1}+L_{i,t}^{d}+L_{i,t-1}} \end{array}$$ (15)

The leverage ratio is used as a proxy to evaluate the risk level of the firm when applying for funding. In our credit-based ARIO, we simulate three models. The first model assumes that banks prioritize economic recovery. In this case, banks are not concerned about the risk levels of firms and lend to them to reduce the indirect demand shortage effect of the negative shock. The second simulated model assumes that banks are risk averse and fund those firms below a limit set on the leverage ratio, λ_*i*,*t*_ ≤ λ. The third model requires third-party intervention; banks are risk averse, as in the second model, but when there is a loan shortage for risky firms, the government or an insurance company provides the necessary funding to avoid and indirect demand shortage effect from the negative shock. We do not discuss the mechanisms of the third-party intervention in this paper: its role is solely to fund risky firms to support economic recovery.

### The recovery process of damaged firms

In the credit-based ARIO model, we assume that directly damaged firms recover by using a reconstruction loan $L_{i,0}^{c}$ that has a maturity *T*_*c*_. First, we assume that all directly damaged firms in the aftermath of the negative shock secure reconstruction loans from their banks. Recall that the direct damage is modelled as a loss to the initial production capacity in the amount of *δ*_*i*,0_. We suppose that the amount of the reconstruction loan is equal to the lost production capacity, which is given by:

$$\begin{array}{c} L_{i,0}^{c}=\delta_{i,0}\times P_{ini,i} \end{array}$$ (16)

The recovery is modelled as a reduction in the magnitude of the damage over time, where:

$$\begin{array}{c} \delta_{i,t}=\left(1-\gamma_{i,t}\right)\times \delta_{i,t-1} \end{array}$$ (17)

*γ*_*i*,*t*_ is the recovery factor and defined in our model based on the financial health of the directly damaged firms. Let $L_{i,t}^{c}$ be the remaining reconstruction loan of firm *i* on day *t*, i.e., the remaining loan after each period’s reimbursement, as discussed in the balance sheet dynamics section. The recovery factor is calculated as follows:

$$\begin{array}{c} \gamma_{i,t}=\frac{D_{i,t}}{L_{i,t}^{c}} \end{array}$$ (18)

Eq 18 reflects that the recovery of firm *i* is faster when it has more deposits (the recovery of production) and when the amount of the reconstruction loan decreases through regular daily payments to banks. This assumption is motivated by the fact that a firm becomes more financially robust when its deposits increase, which facilitates reconstruction in the event of a natural disaster or the resolution of financial resource constraints in the event of a financial crisis. The recovery factor *γ*_*i*,*t*_ is linearly rescaled into the interval [*γ*_*min*_, *γ*_*max*_] ∈ [0, 1]^2^. The values of *γ*_*min*_ and *γ*_*max*_ are calibrated by simulation experiments, as discussed below, i.e., higher values of [*γ*_*min*_, *γ*_*max*_] imply a faster recovery.

### The updating of firm balance sheets

After trading on day *t*, all firms update their balance sheets. First, firms calculate their gross profits given as a fraction of their realized sales:

$$\begin{array}{c} \pi_{i,t}^{G}=\alpha_{i}\times Y_{i,t} \end{array}$$ (19)

The gross profit represents the total sales after paying production expenses, such as technology or labour costs. *α*_*i*_ is the ratio of the gross profit to total sales for firm *i*. Then, for each period until maturity, firms make payments on all the loans accumulated post shock *L*_*i*,*t*_ − *L*_*i*,0_. For example, suppose that firm *i* has *N* loans *j* ∈ 〚1, *N*〛: $L_{i,t}^{j}$ with interest rate $r_{i,t}^{j}$ and maturity *T*. We assume a linear constant amortization of the loans, which yields the following periodic payment:

$$\begin{array}{c} M_{j}=\frac{L_{i,t}^{s,j}\times r_{i,t}^{j}}{1-{\left(1+r_{i,t}^{j}\right)}^{-T}},\;j\in 〚1,N〛 \end{array}$$ (20)

The periodic amount *M*_*j*_ is formed by interest and capital; *M*_*j*_ = *I*_*j*_ + *κ*_*j*_, where $\kappa_{j}=\frac{L_{i,t}^{s,j}}{T_{s}}$. Accordingly, the stock of loans in the balance sheet of firm *i* is updated as follows:

$$\begin{array}{c} L_{i,t+1}=L_{i,t-1}+L_{i,t}^{d}-\sum_{j}\frac{L_{i,t}^{s,j}}{T_{s}}\times I\left(L_{i,t}^{s,j}\right)-\frac{L_{i,t}^{c}}{T_{c}}\times I\left(L_{i,t}^{c}\right) \end{array}$$ (21)

where $\text{I}({\text{L}}_{\text{i},\text{t}}^{\text{s},\text{j}})$ is an indicator function of whether the current loan is paid, i.e., if paid, the value is equal to 0, and 1 otherwise. If a firm cannot pay its loan over consecutive periods *T*^default^, the loan is considered nonperforming until it can be repaid again. The firm continues its activity and accumulates additional wealth to recover and repay its unpaid loans. After paying the banks, each firm calculates its profit as follows:

$$\begin{array}{c} \pi_{i,t}=\pi_{i,t}^{G}-\sum_{j}M_{j} \end{array}$$ (22)

Accordingly, deposits are updated as follows:

$$\begin{array}{c} D_{i,t+1}=D_{i,t}+\pi_{i,t} \end{array}$$ (23)

Finally, equity capital is updated by:

$$\begin{array}{c} E_{i,t+1}=OA+D_{i,t+1}-\left(OL+L_{i,t+1}\right) \end{array}$$ (24)

## Simulation experiments and model validation

Our aim is to validate the proposed credit-based ARIO model. First, we discuss model input validation. Then, we focus on model output validation. See [37] for a deep discussion of the validation approaches in agent-based models.

### The validation of the model inputs

#### The behavioural rules of the credit-based ARIO model

The behavioural rules are one of the most important inputs of our model. They include equations and all assumptions. We rely on previous works to validate them. Tables 3 and 4 in Appendix B offer a summary of the foundations of our implemented behavioural rules.

#### Initialization of the credit-based ARIO model

We estimate the volume of intermediate goods between suppliers and customers as in [14]. The procedure consists of two steps. The first step provides a tentative volume of intermediate goods. Each supplier’s sales are divided among its customers in proportion to their sales. Using the 2016 IO table for Japan, the second step allows us to obtain an aggregate tie-level volume per sector equal to the IO table information. First, we aggregate the tentative volume of intermediate goods at the sector level, and we multiply the obtained total flow to obtain the real exchange value between sectors as in the IO table. Then, the IO table displays the amount of final goods sold from each sector to final consumers (households). The amount of final goods per sector is distributed among all firms from that sector. Accordingly, each supplier *i* sells intermediate goods to its customers *j* denoted by *A*_*ji*_ and final goods to its final consumer denoted by *C*_*i*_. Therefore, the initial production (total sales) of firm *i* is given by the following equation:

$$\begin{array}{c} P_{ini,i}=C_{i}+\sum_{j}A_{ji} \end{array}$$ (25)

We suppose that the balance sheet of firm *i* is given by the following dynamics:

$$\begin{array}{c} OA+D_{i,t}=OL+L_{i,t}+E_{i,t} \end{array}$$ (26)

where *OA* and *OL* are the other assets and other liabilities, respectively, which are assumed to be constant in the model motivated by the short-term post-shock dynamics. *D*_*i*,*t*_ and *L*_*i*,*t*_ are the total deposits and loans of firm *i* on day *t*, where *D*_*i*,*t*_ = ∑_*b*_ *D*_*ib*,*t*_ and *L*_*i*,*t*_ = ∑_*b*_ *L*_*ib*,*t*_ are respectively the sum of deposits and loans of firm *i* with bank *b* on day *t*. *E*_*i*,*t*_ is the equity capital of firm *i* on day *t*. To initialize the firms’ balance sheets, we define the sales multiplier $\mu_{i}^{S}=P_{ini,i}/Sales_{i}^{BS}$, where $Sales_{i}^{BS}$ represents the total sales of firm *i* from its real profit and loss (PL) statement in 2016. Then, the deposits, loans and equity capital from the balance sheet of each firm are multiplied by the sales multiplier to define Eq 26 (*OA* and *OL* are initialized by satisfying the fundamental accounting Eq 26).

The final step of the calibration of the initial values of the model is to weight the bank-firm network links. From the real data set, the bank-firm network is weighted by the values of the loans. We define the loan weight for each firm $\mu_{i}^{L}=Loan_{ib}/Loan_{i}^{BS}$, where *Loan*_*ib*_ is the real amount of total loans from bank *b* to firm *i* given in the bank-firm network in 2016, and $Loan_{i}^{BS}$ is the loan amount in the liabilities statement of firm *i* in 2016. The initial loans and deposits for each bank considered in the model are the result of multiplying the loan weight by the total loans and total deposits estimated in Eq 26. We assume that firm *i* has an initial deposit in bank *b* proportional to its initial loan with that bank.

Based on Eq 19, for each firm *i*, the rate *α*_*i*_ is calculated as the average over 5 years (2011—2016) of the real gross profit to total sales based on the PL statements.

#### Design of experiments and exploration of the parameter space

Table 1 shows the ranges of the parameters in our model. *n* is the average number of days that inventory is held. The number of days for each firm is generated as a Poisson distribution of the average *n* instead of constant. This parameter also requires empirical support, if it is possible as future developments. We assume that firms cannot exceed 1 month (30 days) of inventory (goods could be perishable) and cannot take a substantial production risk by holding inventory for fewer than 10 days. The magnitude of the initial damage *δ*_*i*,0_ can be small, 10% of initial production losses, or very large, 100% of initial production losses. Through simulation experiments, the values of *γ*_*min*_, *γ*_*max*_ are chosen between 0.001 for a slow recovery and 0.055 for a fast recovery. For values less than 0.001, the economy may not recover. When values higher than 0.055, the economy will recover much faster than we observe in Fig 1. When banks are risk managers, they could be very risk averse, λ = 2%, or have a relaxed risk policy by allowing firms with 30% leverage to obtain loans. The maturity of short-term loans used to purchase intermediate goods varies between 1 and 2 months, while the reconstruction loans are longer term and can have a maturity extending to the end of the year after the shock. Throughout the following simulations, we assume that 10% of the firms are initially damaged.

**Table 1:** The possible values of the main parameters of the proposed credit-based ARIO model.

Parameters	Values	2008 Fin. crisis	2011 earthquake
*n*	〚10, 30〛	29	19
*δ*_*i*,0_	[0.1, 1]	0.51	0.95
[*γ*_*min*_,	[0.001: 0.045,	0.001	0.015
*γ*_*max*_]	0.003: 0.055]	0.004	0.025
λ	[0.02, 0.3]	0.05	0.05
*T*_*s*_	〚30, 60〛	53	53
*T*_*c*_	〚300, 400〛	399	399

The calibrated values are defined using the DoE procedure.

Although we have 7 main parameters, the model space is quite large. We use the approach called the design of experiments (DoE) introduced in [38]. The DoE uses Latin hypercube sampling, which was introduced by [39], and indicates how to vary the parameters in a complex simulation model to capture the best response of the system; see [24] for a recent application. For a simulation model with 7 parameters, we need at least 33 samples, as suggested in [24, 38]. To cover the maximum number of points in our model space, we simulate the model for 200 sample combinations of our set of parameters.

### The validation of the model output

Based on the exploration of the parameter space, we have 200 combinations of the considered 7 parameters. Each combination is simulated 1000 times. All simulations are conducted using independent parallel computing on the K supercomputer to reduce the run time (The K computer is the first 10-petaflop supercomputer; it was developed by RIKEN and Fujitsu under a Japanese national project. The system includes 82,944 compute nodes connected by Tofu high-speed interconnects. For further details, see [40]).

#### Calibration of the credit-based ARIO and reproduction of the real IIP of Japan

Let $\Omega^{s_{1}}=\{n^{s_{1}},\delta_{i,0}^{s_{1}},\gamma_{min}^{s_{1}},\gamma_{max}^{s_{1}},T_{s}^{s_{1}},T_{c}^{s_{1}}\}$ be the set of parameters used in the experiments *s*_1_. After 1000 simulations with different random seeds for design *s*_1_, we calculate its simulated VA and define its daily percentage of the initial VA as $\beta_{VA_{s_{1},t}}=\frac{VA_{s_{1},t}}{VA_{s_{1},0}}$. To calibrate our model based on the explored parameter space, we look at minimizing the distance between the simulated and the real output by using the Japanese IIP. As a distance measure, we employ the generalized subtracted L-divergence (GSL-div) introduced by [41], which measures the degree of similarity between the temporal series produced by the model and the real temporal dynamics.

To compare the impact of indirect production losses on risk in the banking system, we assume that banks follow the same policy in 2008 and 2011. Thus, we calibrate, first, the parameters of the system based on the 2011 Great Earthquake. These results are reported in the last column of Table 1. Therefore, the calibration of the credit-based ARIO for the period after the Lehman brothers bankruptcy uses the same values for the leverage ratio λ and loan maturities *T*_*s*_ and *T*_*c*_. Only the production parameters are sampled based on the DoE procedure, and the model is calibrated based on 200 simulation experiments, i.e., the same calibration procedure explained previously. The third column of Table 1 reports the parameter values that reproduce the short-term economic dynamics after the 2008 financial crisis.

The credit-based ARIO model is calibrated using the third lending policy model (see the previous section). Here, we assume that banks are risk managers and consider the leverage ratios of firms through the limit value λ. In addition, when firms cannot obtain loans, they receive exogenous funding at the required amount from a third party. The reason for this choice is explained in the next section.

**Fig 5:**
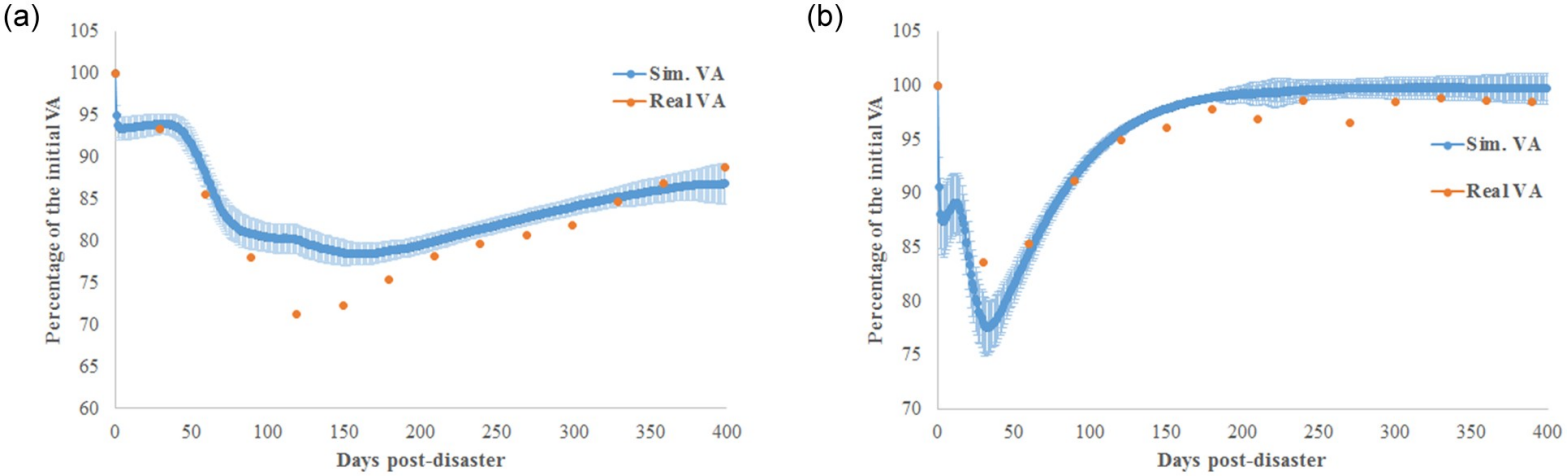
Comparison between simulated and real value added of the Japanese economy. The case of the 2008 Lehman Brothers bankruptcy (a) and the case of the 2011 Great Earthquake (b).

Table 1 shows the calibrated parameters of the model obtained by minimizing the GSL-div measure. Then, based on these parameters, Fig 5 reproduces the dynamics of the Japanese economy after the 2008 Lehman Brothers bankruptcy (Fig 5(a)) and the 2011 Great Earthquake (Fig 5(b)). The main differences between the two crises are as follows: the inventory strategy of firms *n*, the magnitude of the initial damage *δ*_*i*,0_, and the capacity of firms to recover *γ*_*min*_, *γ*_*max*_. During the 2008 financial crisis, the initial damage is much smaller; a natural disaster reaches its maximum damage after 40 days. Moreover, during financial crisis, firms are more risk averse. They are aware that a crisis exists, and they improve their strategy by holding inventory for longer. In contrast, a natural disaster, especially an earthquake, is a surprise, which is why firms employ a softer inventory policy. Finally, after a natural disaster, the recovery is much faster. In fact, the effect of the 2008 financial crisis is delayed: it has lower initial damage but a longer recovery time.

**Fig 6:**
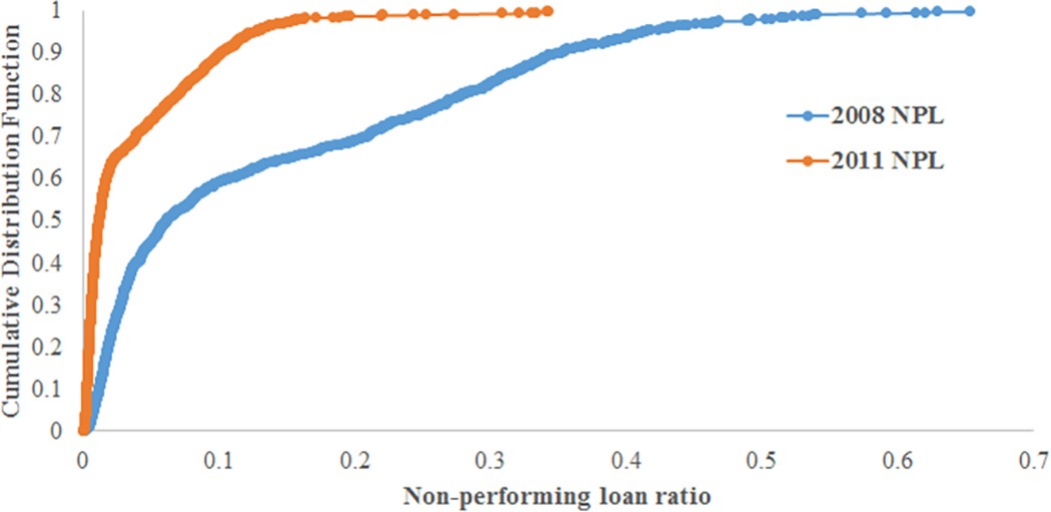
The generated defaulted loans after negative shocks. A comparison between the 2008 Lehman Brothers bankruptcy and the 2011 Great Earthquake cases. The figure compares the CDFs of the percentage of the additional NPL.

Fig 6 compares the cumulative distribution functions (CDFs) of the generated NPL defined as the ratio of defaulted loans to current loans. The NPL rate increases more after the 2008 financial crisis than after the 2011 natural disaster. On average, after the natural disaster, the simulated NPL increased by 3.5%, against 13.5% post financial crisis. Based on the real data depicted in Fig 3, after the 2011 Great Earthquake, the ratio of PLL to total loans increased by 1.2% on average over 1 year. However, the ratio increased by 9.5% on average after the 2008 Lehman Brothers bankruptcy.

#### Sensitivity analysis of the model

[24] discuss the kriging-based approach to analysing the sensitivity of ABMs through the exploration of parameter space using the DoE. This approach interpolates the sampled points to obtain a smooth representation of the parameters and to overcome the issue of ordinary least squares regression (OLS) with low frequency observations. In our exploration of parameter space, we sampled 200 combinations that give us smooth variation of the parameters and large time-scale observations. Therefore, we hereafter use the OLS regression of the following second-order polynomial model presented in [24] to analyse the sensitivity of the credit-based ARIO model:

$$\begin{array}{c} Y\left(x\right)=\beta_{0}+\sum_{g=1}^{k}\beta_{1,g}x_{g}+\sum_{g=1}^{k}\beta_{2,g}x_{g}^{2}+\sum_{g=1}^{k}\sum_{h>g}\beta_{3,g}x_{g}x_{h}+\epsilon \end{array}$$ (27)

*Y*(*x*) is the simulated VA, $x_{g},x_{g}^{2}$ and *x*_*g*_ *x*_*h*_ are the first order, second order, and combined order parameters, respectively, and *β*_*i*,*g*_ are the model coefficients to be estimated. All results of the OLS estimation are in Table 5 in Appendix C. At the first and second order, the model dynamics are influenced only by the magnitude of the initial damage *δ*_*i*,0_. Thus, the observed total losses in the economy are mainly related to the initial intensity of the negative shock. Then, in a second level, the total losses in the economy depend on other factors expressed via the combined effect of the magnitude of the initial damage and other parameters. In fact, the recovery process modelled with *γ* significantly influences the total economic losses post shock (*γ* × *δ*_*i*,0_). Moreover, the inventory policy of firms based on the number of days *n* may influence the total output losses as expressed by the variable *n* × *δ*_*i*,0_. The maturity of short-term loans after the negative shock may also be an instrument to mitigate the effects of the damage (*T*_*s*_ × *δ*_*i*,0_). Finally, the credit-based ARIO model shows that the financial policy post-damage may affect the recovery and the total output losses via the variable *γ* × λ. With the sensitivity analysis in Table 5, we show that the total losses depend first on the magnitude of the damage. Then, the financial support of the banking system and the recovery process could mitigate or aggravate the total output losses.

## Analysis of the effects of negative shocks on the economy using the credit-based ARIO model

We first study the effect of the bank lending strategy on economic recovery. Then, we study the effect of a financial crisis simulated on different Japanese industrial sectors using the calibration of the 2008 financial crisis. Finally, we simulate natural disasters with the calibration of the 2011 Great Earthquake on different prefectures to compare the risks based on geographic location.

### Analysis of the effect of different bank lending models on economic recovery after negatives shocks

The three models described previously are compared below. The results are the outcomes of 1000 simulations with different random seeds. Simulations are performed using the parameters for the 2011 Great Earthquake shown in Table 1. The results are compared on the basis of VA losses, the generated NPL and the liquidity ratio (total loans over total deposits).

**Fig 7:**
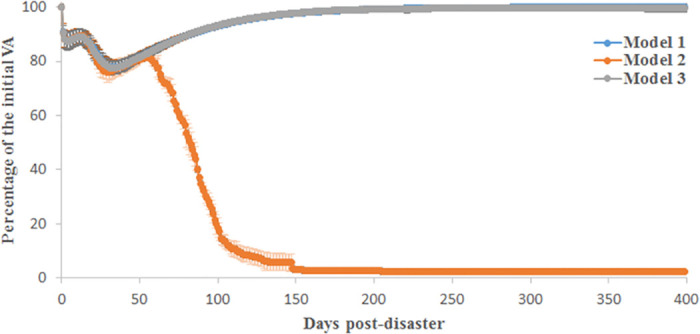
The value-added losses after a negative shock. Three models are simulated based on the 2011 Great Earthquake parameters. Outcomes from models 1 and 3 are very similar, and we cannot distinguish between the two curves in the plot.

**Fig 8:**
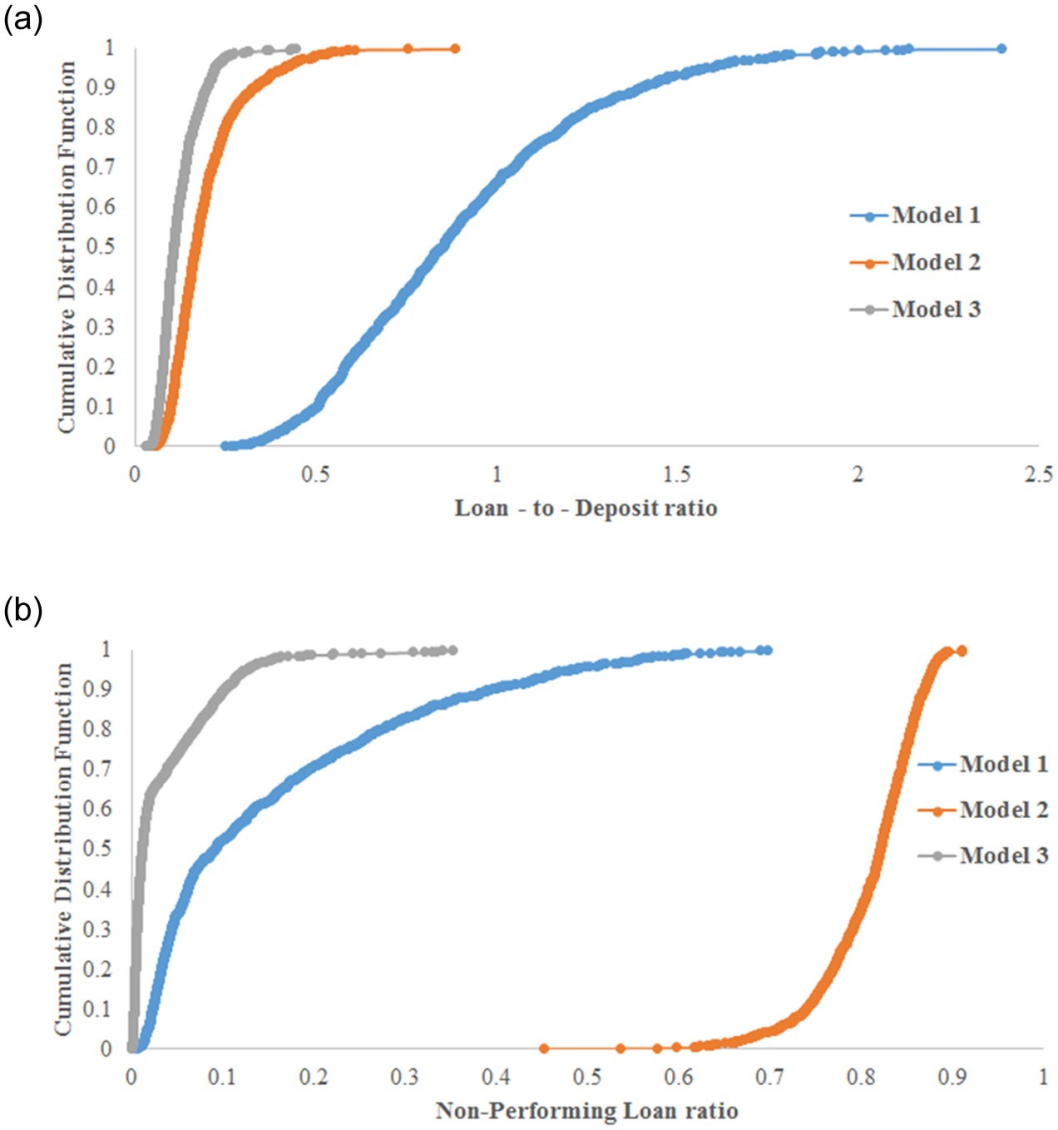
The simulated financial losses after a negative shock. Three policies are analyzed based on the 2011 Great Earthquake parameters. (a) The evolution of the liquidity ratio for banks; (b) the generated non-performing loans.

After a negative shock and due to supply shortages, wealth accumulation decreases, and firms need loans to produce and survive. Accordingly, when banks follow a risk-averse policy (model 2), some firms cannot continue production because they cannot purchase intermediate goods due to their high leverage. Then, the economy is damaged by a second, loan-related wave of the crisis and bottoms out 70 days after the initial negative shock; see Fig 7. In model 2, banks seek to minimize their risk. The liquidity ratio is successfully minimized, in contrast to model 1, where banks do not follow a risk-averse strategy; see Fig 8(a). However, Fig 8(b) shows that the NPL rate increases drastically in model 2. In fact, firms were initially in good financial condition, had low leverage and could secure loans. Then, when banks stop making loans because firms were highly leveraged, the overall economy is significantly damaged, and firms default on past loans, which then remain unpaid. Model 1 is also problematic despite the fact that NPL growth is not very high. Banks supply a high volume of loans compared to what they can earn as deposits because production decreases after the negative shock. This situation is too risky for banks because it places them in a liquidity disequilibrium, which affects their asset and liability management and could lead to a serious financial crisis. See [42, 43]; i.e., in the real world, the liquidity issue is closely related to the function of the central bank (we intentionally do not introduce a central bank in this model). Model 3, as shown in Figs 7 and 8, allows for the complete recovery of the economy, the maintenance of a low liquidity ratio (lower than that in model 2) and low NPL growth (lower than that in model 1). Accordingly, banks must collaborate with other institutions, such as insurance companies and governmental institutions, to fund firms during the first year after the shock to boost recovery and maintain a low level of financial risk. In all experiments hereafter, model 3 is used with the calibration given in Table 1.

### Economic losses when varying the initially damaged industrial sectors

We consider 8 sectors: chemical and petroleum manufacturing; machinery manufacturing; plastic, metal and ceramic product manufacturing; food manufacturing; construction; transport; wholesale trading and retail trading. We simulate the initial damage to the industrial sectors using the 2008 financial crisis parameters (see Table 1). The results present the impact of the initial damage to the entire economy as the average of 100 simulations with different random seeds.

**Fig 9:**
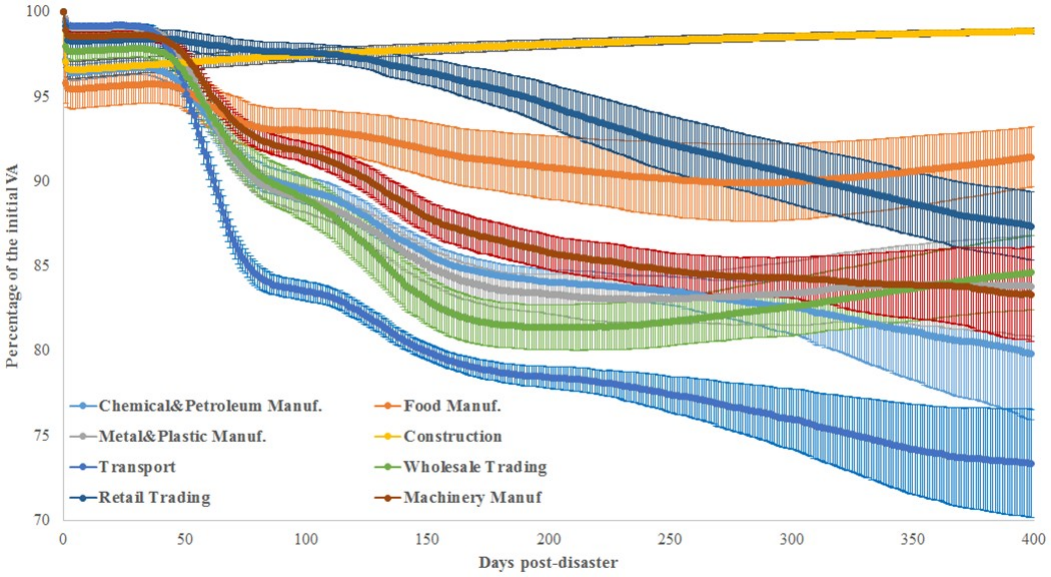
The post-crisis losses in value added simulated using the 2008 financial crisis parameters. Initially, damaged firms are chosen at random from one industrial sector. The 8 major industrial sectors in the production network of Japanese listed firms are considered.

Fig 9 indicates that the total economic losses differ based on which industrial sectors are initially damaged. Little damage is observed when the construction sector is initially hit by a negative shock, i.e., non-significant indirect losses and a rapid economic recovery. When a crisis initially hits food manufacturing or retail trading, the economic losses are also limited in size, i.e., a maximum of 12% of the initial VA is lost. However, significant losses are observed when the following sectors are initially damaged: chemical and petroleum manufacturing; machinery manufacturing; plastic, metal and ceramic product manufacturing; and wholesale trading. Finally, the largest impact is observed when the transport sector is initially damaged; the economy loses up to 30% of its production capacity over 1 year.

**Fig 10:**
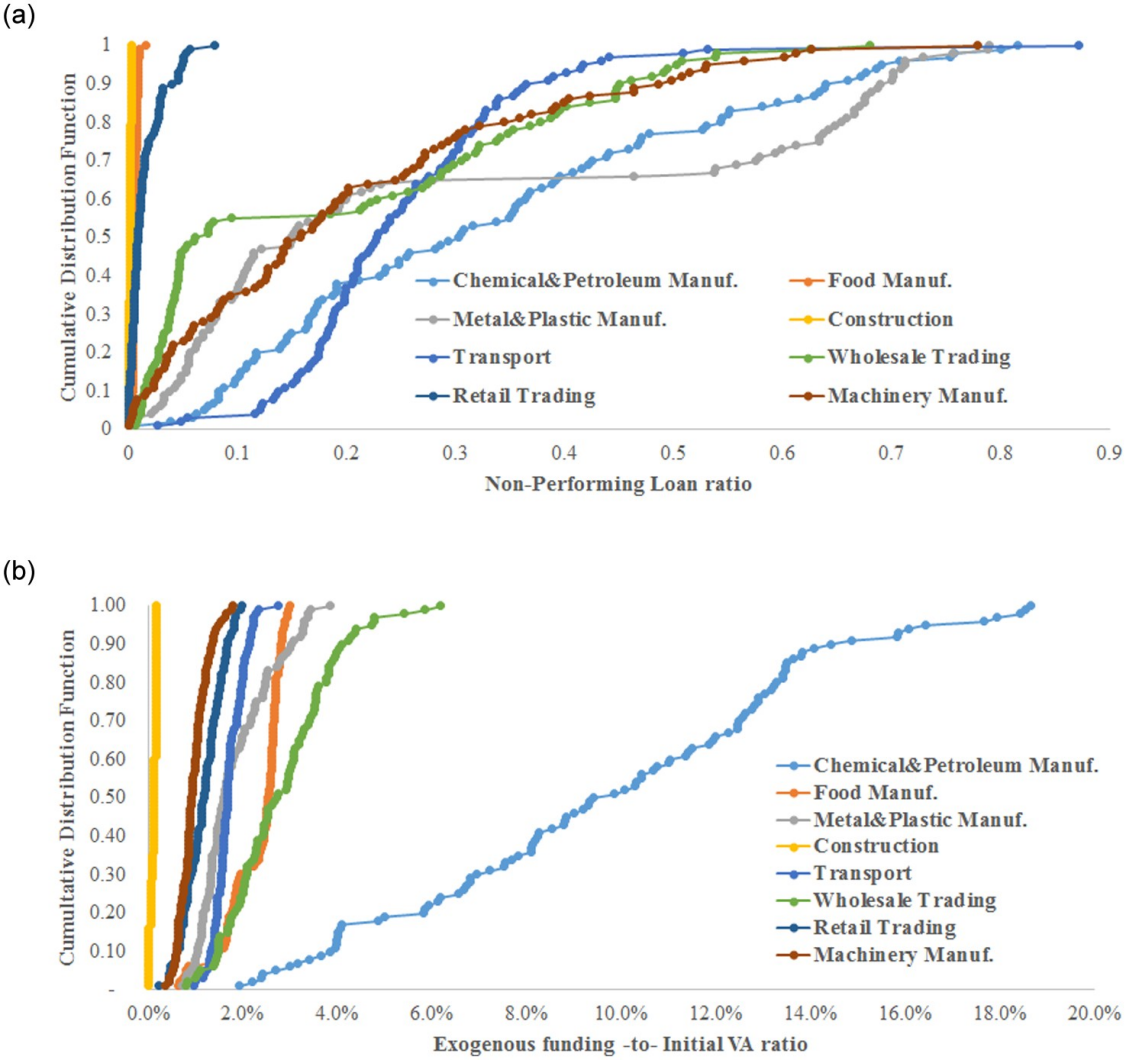
The post-crisis financial losses simulated using the 2008 financial crisis parameters. Initially, damaged firms are chosen at random from one industrial sector. The 8 major industrial sectors in the production network of Japanese listed firms are considered. Graphs show the generated non-performing loans (a) and the necessary exogenous funding under policy 3 required for the economy to recover (b).

In terms of NPL, in Fig 10(a), industrial sectors can be placed into two categories. The first is industrial sectors with a limited impact on the banking system: construction, food manufacturing and retail trading (sectors with lower economic losses), i.e., when firms from these sectors are damaged initially, the banking system suffers fewer defaulted loans. The second category is industrial sectors with significant impacts on the banking system: all other sectors generate high NPL, as summarized in Table 2, i.e., column 2 shows the average NPL generated in this case. The exogenous funding needed by firms is calculated as a part of the initial VA generated by the economy, and the CDFs are displayed in Fig 10(b). When the construction sector is damaged, the economy requires the least exogenous funding (less than 0.1% of the initial VA) to recover. However, when firms from the chemical and petroleum industries are damaged initially by the effects of a financial crisis, the required amount of exogenous funding is the highest: 9.7% of the initial VA of the economy on average, and it could reach 18.6% of the initial VA of the economy.

**Table 2:** Properties of the initially damaged sectors.

Sectors	NPL	Ex. funding	Initial VA	In–strength	Out-strength	Degree	Clustering	K_nn_
Chemical&Petroleum manuf.	33.1%	9.7%	36,396	74.9	142.4	802	0.41	33.1
Wholesale trading	18.8%	2.8%	32,075	17.9	50.3	2,849	0.15	21.0
Machinery manuf.	20.4%	1.0%	28,714	22.7	15.8	1,628	0.32	29.9
Food manuf.	0.6%	2.3%	23,814	25.9	14.9	322	0.24	27.5
Construction	0.1%	0.1%	19,595	32.2	0	627	0.16	26
Retail trading	1.5%	1.2%	17,698	64.2	0.3	598	0.21	24.5
Plastic, metal&ceramic manuf.	29.5%	1.3%	12,960	33.3	30.8	1,567	0.37	33.2
Transport	25.1%	1.7%	4,765	14.6	13.8	505	0.48	38.9

Columns 2 and 3 report the average of the simulated generated NPL and exogenous funding to initial value added. Industrial sectors are classed by their value added in column 4. Columns 5 and 6 show the average in-strength and out-strength of each sector. Column 7 shows the total unweighted degrees. Columns 8 and 9 report the clustering coefficient and the K_nn_ of each sector in the sector-based production network.

Table 2 reports the properties of each industrial sector to explain their systemic risk. Sectors are classified in Table 2 in descending order with respect to their initial VA; see column 4 of the table. Columns 2 and 3 report the average of the generated NPL and the exogenous funding required, respectively. Columns 5, 6 and 7 report the average in-strength and out-strength (the sum of money in- and outflows, respectively) and the total unweighted degrees (the sum of all firm-firm links) in each sector. Then, the sector-based production network is considered, i.e., if a firm from sector A is connected to a firm from sector B, a link from sector A to sector B is considered. The sector-based production network is weighted based on the aggregate flow of intermediate goods from our production network of listed firms. Columns 8 and 9 report the clustering coefficient and the average nearest neighbour degrees K_nn_. K_nn_ computes the average degrees of the neighbours of a node in the network. It is useful to know whether a node is connected to highly or less connected neighbours. of each sector in the sector-based production network to specify the centrality of each sector in the economy.

In terms of NPL growth after the crisis, the riskiest sectors are chemical and petroleum manufacturing (33.1%), plastic, metal and ceramic product manufacturing (29.5%), transport (25.1%), machinery manufacturing (20.4%), and wholesale trading (18.8%). The chemical and petroleum manufacturing sector has the highest initial VA. Although the two latter sectors have the lowest initial VA, their impact on the economy leads to a higher growth rate of defaulted loans than the construction sector, which generates only 0.1% of additional NPL despite an initial VA four times higher than that of the transport sector, i.e., 19,595 for the construction sector compared to 4,765 for the transport sector. The riskiest sectors in terms of defaulted loans have a central position in the production network. In fact, some of them supply large amounts of intermediate goods based on out-strength (chemical and petroleum manufacturing, wholesale trading, and plastic, metal and ceramic product manufacturing). Other risky sectors are connected with highly connected sectors (higher K_nn_) and display high clustering coefficients, such as transport and plastic, metal and ceramic product manufacturing. Moreover, we can explain the largest losses to the economy by the initial damage to the transport sector resulting from its central position in the supply chain. With a clustering coefficient of 0.48, this sector forms triangles with other sectors, which accelerates the spillover of the crisis. In addition, K_nn_ = 38.9 implies that the transport sector has, on average, the neighbours with the highest degrees, which stimulates the contagion effect in the event of a crisis. However, as shown in Table 2, sectors such as construction and retail trading have a very low impact on the production network of Japanese listed firms. The construction sector has no customers in the production network. The retail trading sector has few customers in the production network, but its outflow of money (out-strength) is too low. Therefore, initial damage to the construction or retail trading sectors causes a small supply shortage effect. In terms of strength, machinery manufacturing and food manufacturing have similar properties. However, the former has a larger impact on the economy, which is explained by its higher number of partners, 1,628, compared to only 322 for food manufacturing (which has the fewest partners in the production network). Accordingly, by jointly considering the financial and network properties of industrial sectors, we can understand their systemic risk.

### Economic losses when varying the initial damaged geographic locations

**Fig 11:**
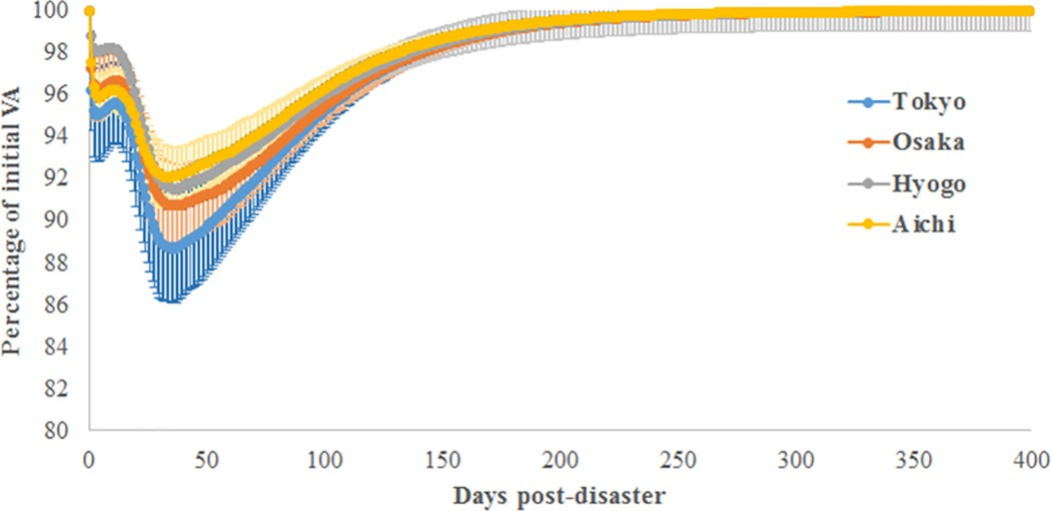
The post-disaster value-added losses simulated using the 2011 Great Earthquake parameters. Initially, damaged firms are chosen at random from one industrial sector. The 8 major industrial sectors in the production network of Japanese listed firms are considered.

Here, we simulate a natural disaster with the properties of the 2011 Great Earthquake. The aim is to identify the most vulnerable Japanese prefectures. First, the economic effect on VA losses is depicted in Fig 11. Most headquarters are located in Tokyo prefecture, and our data consider headquarters locations and not branch locations. To avoid having effects driven by the size of each prefecture (the total number of firms), we consider the same number of initially damaged firms per prefecture, i.e., 4% of the total number of firms in the supply chain.

**Fig 12:**
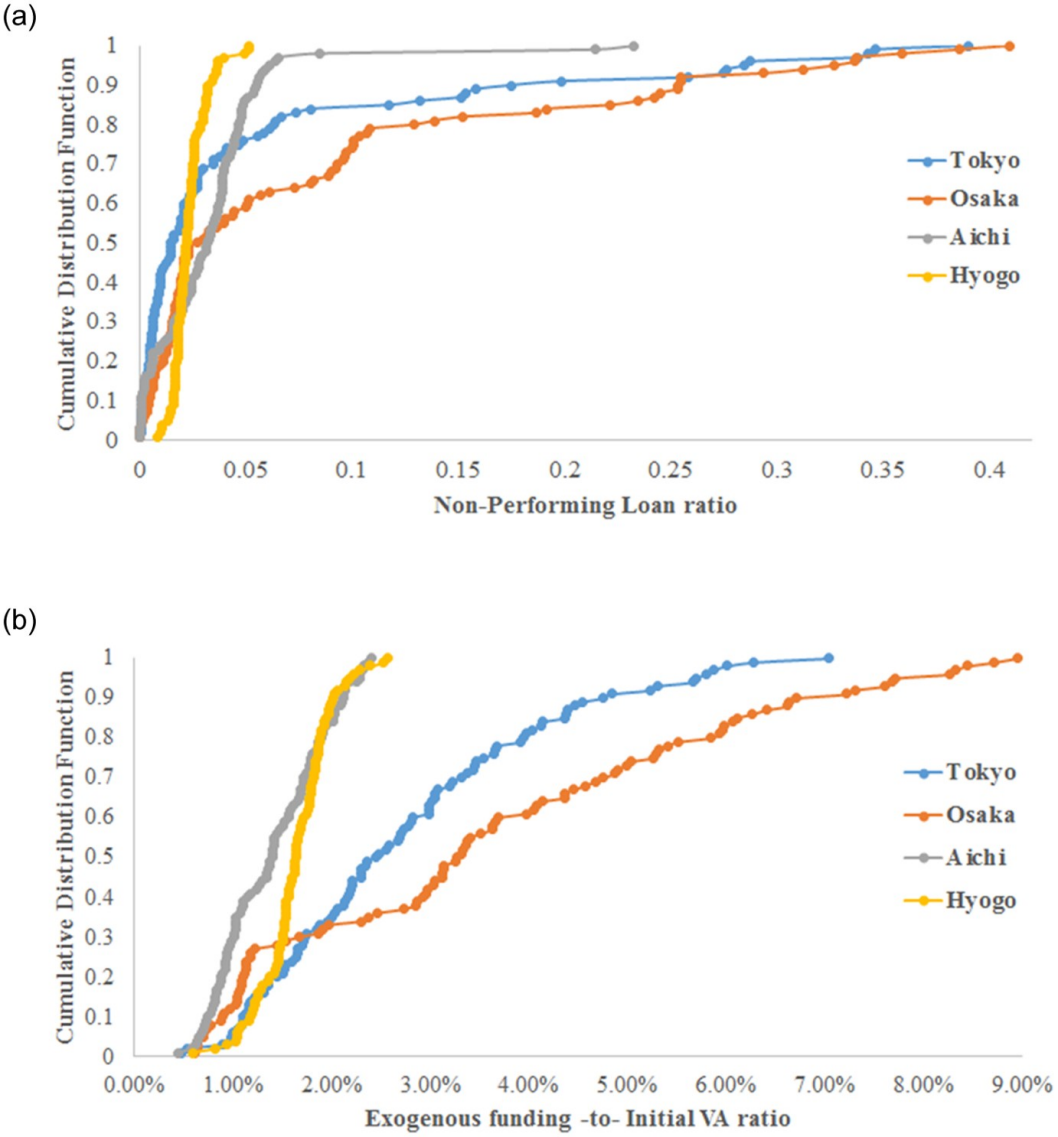
The simulated post-disaster financial losses simulated using the 2011 Great Earthquake parameters. Initially, damaged firms are chosen at random from one industrial sector. The 8 major industrial sectors in the production network of Japanese listed firms are considered. Graphs show the simulated non-performing loans (a) and the exogenous funding under policy 3 required for the economy to recover (b).

As expected, when Tokyo prefecture is directly damaged, the economy faces the highest losses. When Aichi or Hyogo prefectures are initially damaged, a smaller effect on the economy is observed. Fig 12 depicts the financial effects of the simulated natural disaster across Japanese prefectures. In terms of generated NPL (see Fig 12(a)), the effect of initial damage in Aichi and Hyogo prefectures is the lowest, i.e., 3.2% and 2.3%, respectively. This finding is confirmed in Fig 12(b), where the economy requires a small amount of additional exogenous funding to recover when these latter prefectures are initially damaged, i.e., 1.4% and 1.5% for Aichi and Hyogo prefectures, respectively. Although initial damage to Tokyo causes the highest production losses, Fig 12(a) and 12(b) show that damage to Osaka prefecture causes the greatest financial losses.

**Fig 13:**
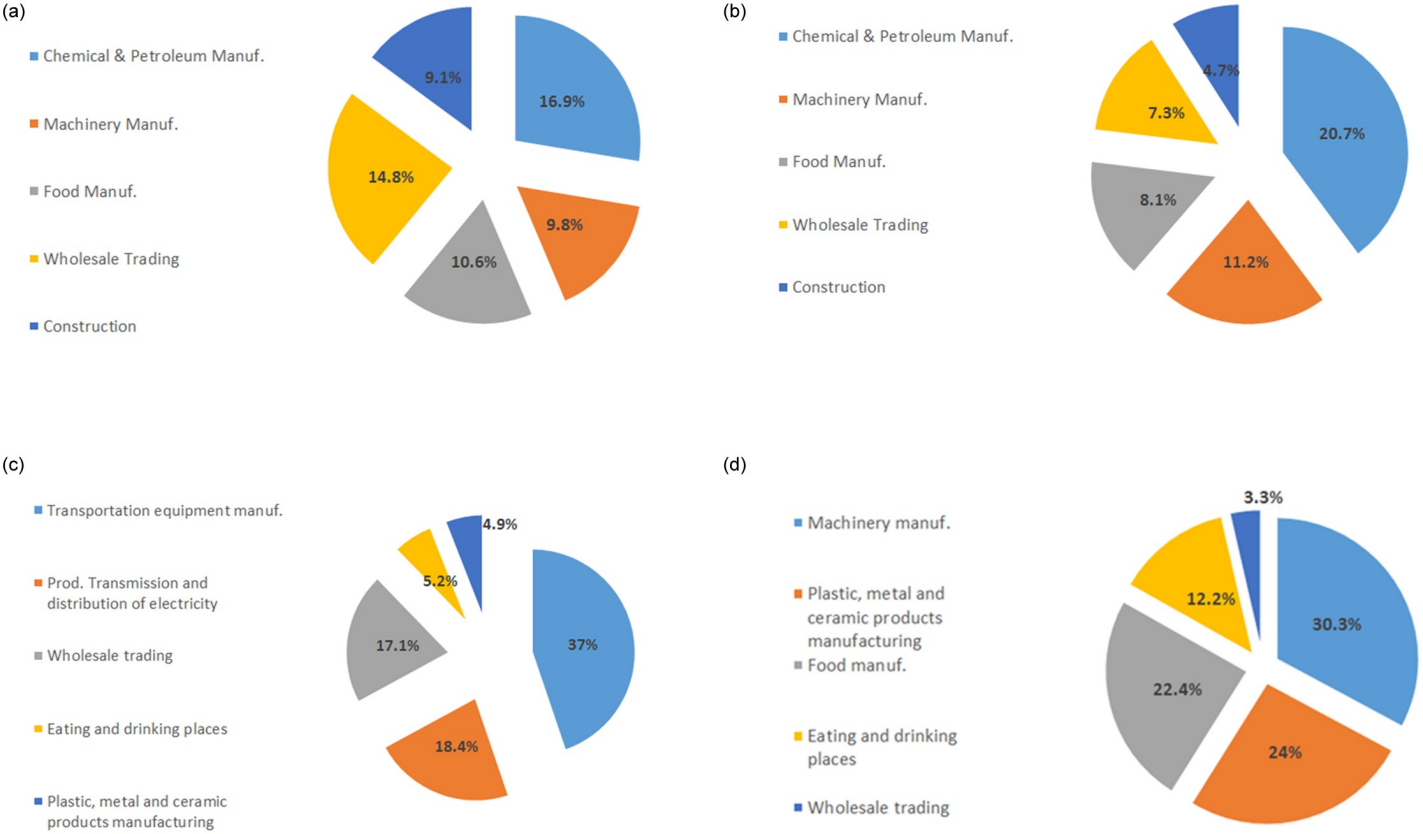
The business structure of the considered Japanese prefectures. Tokyo prefecture (Fig 13(a)), Osaka prefecture (Fig 13(b)), Aichi prefecture (Fig 13(c)), and Hyogo prefecture (Fig 13(d)).

**Fig 14:**
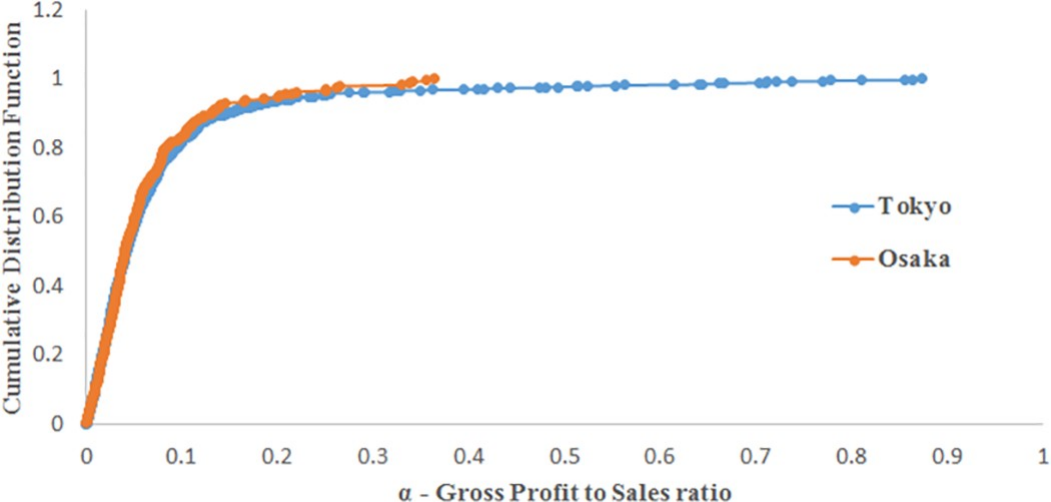
Comparison between the CDFs of the gross profit to sales ratio *α*_*i*_. The case of firms in Tokyo and Osaka prefectures.

To analyse this difference among prefectures, Fig 13 shows the business structure of each prefecture, i.e., the weights of the top 5 industrial sectors in each prefecture. Transportation equipment manufacturing is the dominant industrial sector in Aichi prefecture, as shown in Fig 13(c); Toyota’s headquarters is in Aichi prefecture. This sector causes little damage to the production network because it has only 25 customers, i.e., limited risk of supply shortages post disaster based on our previous analysis. Approximately 35% of firms in Hyogo prefecture are in the food manufacturing and food and beverage services, as shown in Fig 13(d), which are not sectors at high risk of supply shortages (low number of customers in the production network of listed firms). Tokyo and Osaka prefectures have similar business structures (see Fig 13(a) and 13(b)), with a higher weight of chemical and petroleum manufacturing in Osaka prefecture. In terms of VA, Tokyo prefecture contributes 10 times more than Osaka, which explains why we observe the greatest impact on economic losses when Tokyo is initially damaged. Fig 14 represents the CDF of the ratio *α*_*i*_ included in the credit-based ARIO model to calculate the profit of firm *i* after selling its intermediate goods. We recall that this ratio is calculated based on the real PL statements of Japanese listed firms (gross profits divided by total sales); see the explanations in the model section. Fig 14 shows that, on average, firms in Tokyo prefecture generate more profit from their sales than do firms in Osaka prefecture. This implies that firms in Osaka prefecture have lower profitability and that their deposits grow more slowly than the deposits of firms in Tokyo prefecture. Consequently, based on our model, firms in Osaka prefecture will demand more loans, which explains the higher NPL rate and the higher need for exogenous funding there. Thus, although Tokyo and Osaka have similar business structures in terms of industrial sectors, the difference in the financial efficiency of firms explains the higher financial damages faced by the economy when firms from Osaka prefecture are initially damaged.

## Discussion and conclusion

[22] discusses the effect of financial crises and natural disasters, which are recurrent phenomena, on the economy and households’ coping strategies. Although they have different mechanisms, the two types of negative shocks have similar impacts on the economy, i.e., slowed production and greater financial risk, as shown in Figs 1, 2, and 3, and modified household behaviour, as discussed by [22]. In the economic literature, the two types of negative shocks are often considered separately; see [34] for financial crises and [8] for natural disasters. The first contribution of the current paper was to propose a credit-based ARIO framework that permits the simulation of different types of shocks. Using a calibration procedure employing Latin hypercube sampling, we reproduced the economic dynamics of Japan after the 2008 Lehman Brothers bankruptcy and the 2011 Great Earthquake. The firms’ strategies differ between these two negative shocks. During the 2008 financial crisis, firms had prior information about the recession because it began in the U.S. and reached Japan after a delay. The uncertainty faced by firms increased, which is sometimes reflected in a stronger inventory policy, i.e., planning to hold sufficient inventory for a longer period. However, the initial direct damage of the 2011 natural disaster was much stronger. The direct damage to Japan during the 2008 financial crisis was due to the financial resource constraints faced by firms. Thus, production was able to continue, albeit with lower capacity, i.e., there was no material damage to Japanese firms. However, after the 2011 Great Earthquake, the directly damaged firms may have suffered from the destruction of buildings or roads used for transportation, for example. Therefore, production could have halted in the aftermath of the disaster.

During the 2008 financial crisis, the economic recovery was slower. In the credit-based ARIO model, the slow recovery is captured by the calibration of the parameters *γ*_*min*_ and *γ*_*max*_. In reality, the slower recovery from a financial crisis may be due to various causes, such as economic uncertainty or a negative demand shock to the domestic market; see [44]. These mechanisms demand more complex modelling of the recovery, which represents a direction for future research.

We considered three models. The first and second models are harmful to the economy. When banks supply loans without any risk policy, they face serious liquidity problems due to firms’ inability to generate sufficient value added and increase the aggregate deposits in the economy. Moreover, when banks follow a strict risk policy by monitoring firms’ leverage ratios, the economy cannot recover after a negative shock, and banks face substantial amounts of defaulted loans, which dramatically increases their non-performing loans ratio. Therefore, we showed through simulation that the economy needs exogenous funding, expressed as a percentage of the pre-shock value added generated by the economy. Indeed, when risky firms exceed the leverage allowed by banks, intervention by a third party in the form of offering production loans to firms helps the economic recovery, reduces the non-performing loans in the banking system and holds the liquidity ratio (loans to deposits) at a low level. We note that the simulation of the third model may transfer credit risk to a third party. Strategies for credit risk sharing could be addressed in future work.

The credit-based ARIO model was used to understand the systemic risk of the most important Japanese industrial sectors in the supply chain and their impact on the bank-firm network. The model was simulated based on the calibration for the 2008 financial crisis and considered initial damage from a particular sector to measure the effect on the overall economy. The main results showed that industrial sectors are risky because they can have a high outflow of intermediate goods, such as chemical and petroleum manufacturing, or occupy a central position in the supply chain, such as the transport sector. Industrial sectors with low outflows of intermediate goods have a small impact on the economy when they are initially damaged, for example, the construction and retail trade sectors. Then, we estimated the model with parameters calibrated based on the 2011 Great Earthquake to understand the systemic risk of the most important prefectures in terms of industrial weight. Tokyo and Osaka prefectures were identified as the riskiest prefectures; this was explained by their business structures, which are formed by risky industrial sectors such as chemical and petroleum companies and wholesale trade companies. We found that Osaka prefecture causes higher financial damage than Tokyo prefecture because of the lower profit efficiency of its firms, which increases non-performing loans and funding needs when it is initially damaged.

In a nutshell, our study shows that the spread of economic losses in the aftermath of negative exogenous shock depends on four factors, besides the magnitude of the initial shock: i) the centrality of directly damaged firms in the production network, i.e., importance of economic sectors; ii) the firms’ inventory strategies; iii) the recovery capacity of firms; vi) the initial financial condition of firms in the production network.

This work is a first step in the development of ARIO modelling to consider a larger economy and estimate the more complex indirect losses following a negative shock in the short term. The results demand further reflection. First, it is important to consider the reactions of banks to the shock and how they manage their portfolios of defaulted loans and the difficult liquidity conditions to maintain their market efficiency. For this reason, it will be essential to consider interbank linkages in our model. Second, we intend to model the behaviour of households after the negative shock to obtain better estimates of the global indirect losses, which include shocks to final consumption.

## Appendix A: A schematic representation of the credit-based ARIO model

**Fig 15:**
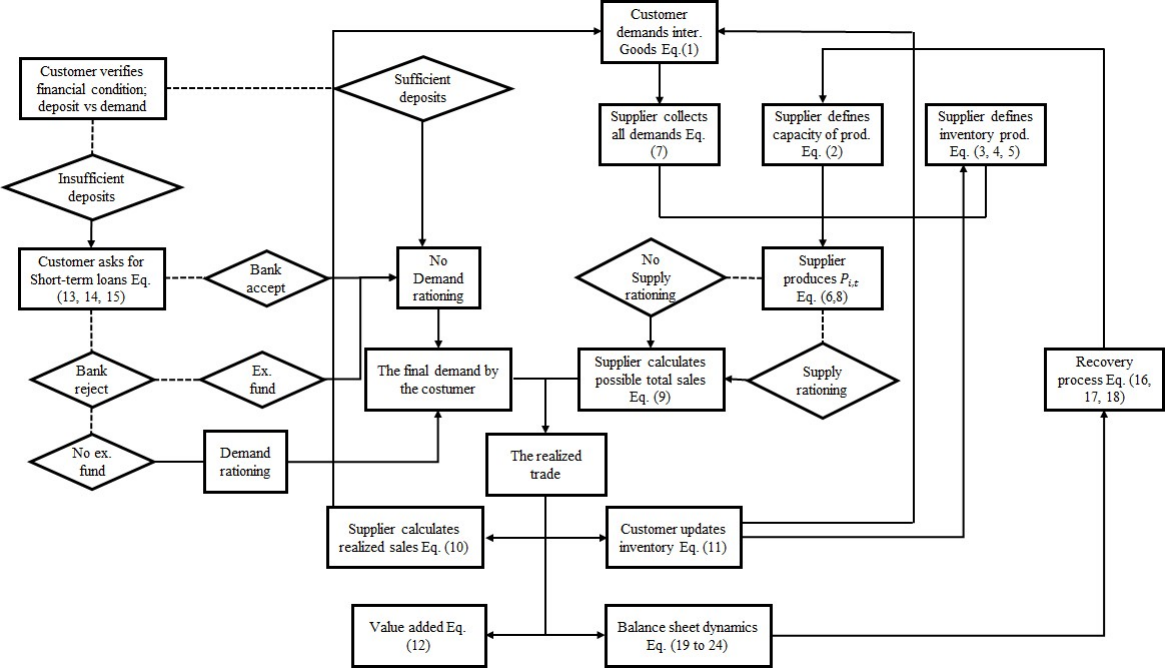
Schematic representation of the credit-based ARIO model. This diagram describes the different model components and their associated equations.

**Fig 16:**
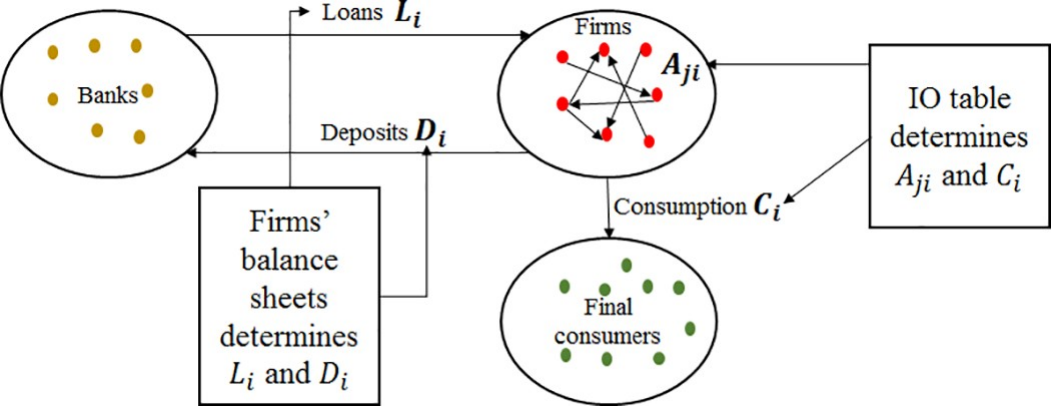
Data structure of the credit-based ARIO model. The figure shows how data of the credit-based ARIO model are constructed from four databases: i) the TSR production network; ii) the Nikkei bank-firm network; iii) the Nikkei firms’ balance sheets; iv) the IO table.

Fig 15 offers a diagram of the credit-based ARIO. It shows the different model components and processes. Each process is related to the equations discussed in the main text of manuscript. More details on the equations are given in Tables 3 and 4. Fig 16 shows the data structure of the credit-based ARIO model.

**Table 3:** The behavioural rules of production in the credit-based ARIO.

Equations	Economic foundation	Literature
Eq 1	Customer *i* decides its consumption of intermediate good *j*, based on its pre-shock levels of consumption and production, its past level of production, its level of inventory of intermediate good *j*, and the number of days its inventory holds.	[11, 13, 14]
Eq 2	Direct damaged suppliers lose their initial production capacity, i.e., initial supply shock.	[11, 13, 14]
Eq 3	Supplier *i* calculates its inventory of intermediate good *s*.	[13, 14]
Eq 4	Supplier *i* determines its pre-to-post shock ratio of inventory of intermediate good *s*.	[13, 14]
Eq 5	Possible production based on intermediate good *s* is the proportion of the pre-shock production in the pre-to-post shock ratio of inventory of intermediate good *s* as calculated in Eq 4.	[13, 14]
Eq 6	Maximum production is the minimum between the production capacity, Eq 2, and inventory-based production, Eq 5.	[13, 14]
Eq 7	Supplier *i* calculates the received demand from all its customers in the supply chain network.	[13, 14]
Eq 8	Production does not exceed the received demand. Although the supplier could recover, the level of production depends also on the recovery of its customers in the supply chain.	[13, 14]
Eq 9	If the received demand by supplier *i*, Eq 7, is higher than its production, Eq 8, its customers from the production network are rationed based on their pre-to-post shock demand ratio. The possible realized sales are equal to production from Eq 8.	[13, 14]
Eq 10	Customers could have a funding gap as explained in [33]. If deposits cannot meet the demand for goods, a customer asks for a loan based on its connections in the bank-firm network. If funding is not available, the customer reduces its consumption, as discussed empirically [15].	[15, 33]
Eq 11	Inventory dynamics: the new inventory is equal to the past inventory plus the purchased goods minus the used goods for production	[13, 14]
Eq 12	The value added is measured as the total output minus the total used input.	[13, 14]

Column 1 presents the behavioural rules through the implemented equations in the model. Column 2 discusses the economic foundation of the behavioural rules. Column 3 refers the literature discussing the behavioural rules.

**Table 4:** The behavioural rules of finance in the credit-based ARIO.

Equations	Economic foundation	Literature
Eq 13	The funding gap as the difference between the level of deposits and the total demand of intermediate goods.	[33]
Eq 14	Risk-based pricing of the interest rate. Firms with lower production have a weaker financial condition and then receive higher interest rates for loans.	[35, 36]
Eq 15	The leverage ratio of firms is defined as the weight of the loans to that of equity.	[33]
Eqs 16 to 18	The recovery process in Eq 17 follows the model of [13, 14]. In our model, we relate the dynamics of the recovery process to the financial condition of damaged firms. When the financial condition is improving, with higher deposits and lower loans, the firm recovers faster.	[13, 14]
Eqs 19 to 24	Balance sheet dynamics and accounting rules.	[33]

Column 1 presents the behavioural rules through the implemented equations in the model. Column 2 discusses the economic foundation of the behavioural rules. Column 3 refers to the literature discussing the behavioural rules.

## Appendix B: The behavioural rules of the credit-based ARIO

Tables 3 and 4 regroup all the equations of the credit-based ARIO model. Table 3 exposes the behavioural rules of the production equations, and Table 4 exposes the behavioural rules of the finance equations.

## Appendix C: Estimation results of the sensitivity analysis

Table 5.

**Table 5:** Sensitivity analysis.

Coefficients	Estimate	Std. Error	t-value	p-value
Intercept	7.83	2.78	2.82	0.0062**
*n*	0.12	0.19	0.61	0.54
*γ*	5.31	14.09	0.38	0.71
*δ*_*i*,0_	13.36	1.26	10.56	0.00***
λ	-5.75	5.10	-1.13	0.26
*T*_*s*_	0.04	0.04	1.13	0.26
*T*_*c*_	0.001	0.006	0.17	0.86
*n*^2^	-0.003	0.004	-0.61	0.54
*γ*^2^	-3.56	8.02	-0.44	0.67
$\delta_{i,0}^{2}$	-4.18	0.46	-8.95	0.00***
λ^2^	-9.46	7.75	-1.22	0.22
$T_{s}^{2}$	-2.22e-04	2.73e-04	-0.81	0.42
$T_{c}^{2}$	-4.13e-06	8.83e-06	-0.47	0.64
*nγ*	-0.66	0.54	-1.21	0.22
*nδ*_*i*,0_	-0.12	0.04	-2.58	0.01*
*nλ*	0.07	0.15	0.47	0.64
*nT*_*s*_	7.91e-05	1.07e-03	0.074	0.94
*nT*_*c*_	1.62e-04	1.73e-04	0.93	0.35
*γδ*_*i*,0_	-28.18	5.16	-5.45	0.00***
*γ*λ	65.84	23.23	2.83	0.005**
*γT*_*s*_	-0.06	0.14	-0.39	0.69
*γT*_*c*_	-0.02	-0.02	-1.00	0.32
*δ*_*i*,0_λ	1.36	1.48	0.92	0.36
*δ*_*i*,0_*T*_*s*_	-0.03	0.01	-2.81	0.006**
*δ*_*i*,0_*T*_*c*_	-1.13e-03	1.65e-03	-0.68	0.49
λ*T*_*s*_	3.20e-03	0.04	0.07	0.94
λ*T*_*c*_	9.79e-03	6.96e-03	1.41	0.16
*T*_*s*_*T*_*c*_	-2.98e-05	4.98e-05	-0.59	0.55

OLS estimation of a second-order polynomial model based on DoE parameter space exploration. (*γ* = mean(*γ*_min_, *γ*_max_)). Significant codes: 0 ‘***’ 0.001 ‘**’ 0.01 ‘*’ 0.05 ‘.’ 0.1. Multiple R-squared: 0.9675, Adjusted R-squared: 0.956.
